# Supplementary material for: Cholinergic neuron-to-glioblastoma synapses in a human iPSC-derived co-culture model
Source: Stem Cell Reports. 2025 Jun 19;20(7):102534. doi: 10.1016/j.stemcr.2025.102534 (PMC12277791; doi:10.1016/j.stemcr.2025.102534)
Supplement: Document S1. Figures S1–S4 and supplemental methods [file mmc1.pdf]

Stem Cell Reports, Volume *20*

Supplemental Information

Cholinergic neuron-to-glioblastoma synapses in a human iPSC-derived co-culture model

Yusha Sun, Xin Wang, Zhijian Zhang, Kristen H. Park, Yicheng Wu, Weifan Dong, Daniel Y. Zhang, Yao Fu, Feng Zhang, Zev A. Binder, Emily Ling-Lin Pai, MacLean P. Nasrallah, Kimberly M. Christian, Donald M. O'Rourke, Nicolas Toni, Guo-li Ming, and Hongjun Song

Cholinergic neuron-to-glioblastoma synapses in a human iPSC-derived co-culture model

Yusha Sun, Xin Wang, Zhijian Zhang, Kristen H. Park, Yicheng Wu, Weifan Dong, Daniel Y. Zhang, Yao Fu, Feng Zhang, Zev A. Binder, Emily Ling-Lin Pai, MacLean P. Nasrallah, Kimberly M. Christian, Donald M. O'Rourke, Nicolas Toni, Guo-li Ming, and Hongjun Song

Supplementary Information Inventory

Figures S1-4

Tables S1-3

Supplementary Methods

SUPPLEMENTARY FIGURES

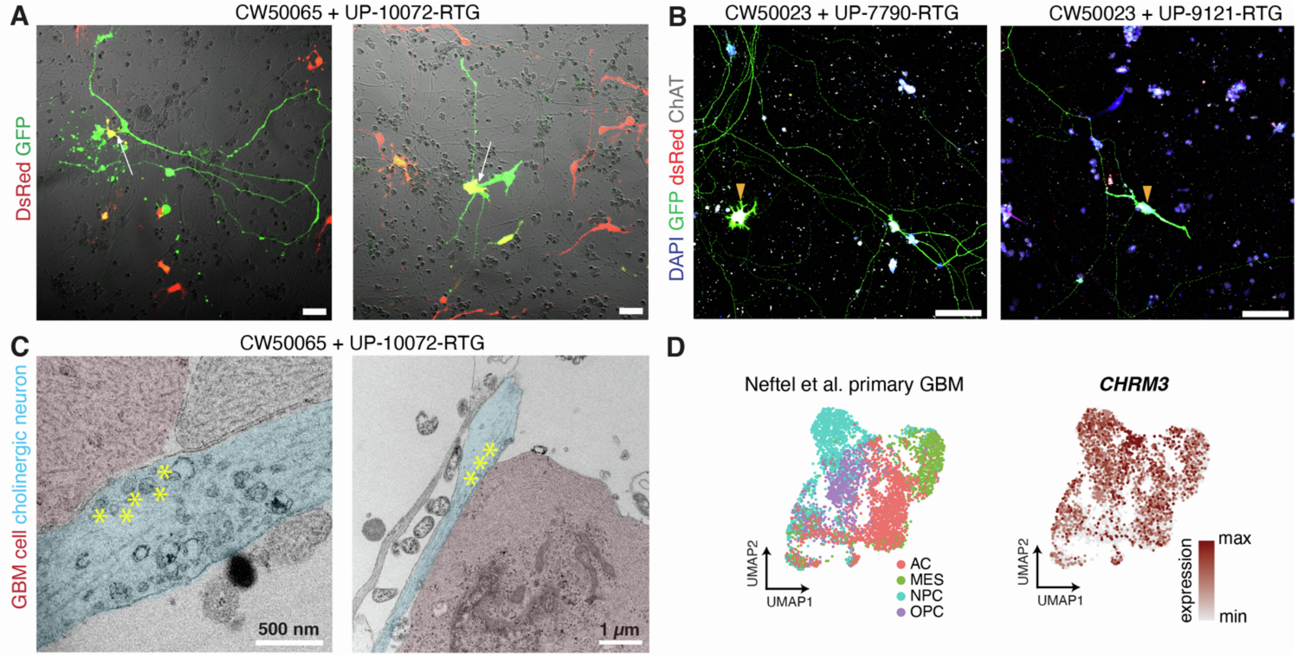

Figure S1. Extended characterization of human cholinergic neuron-to-glioblastoma synapses, related to Figures 1 and 2.

(A) Sample brightfield microscopy images overlaid with fluorescent channels at 2 days post co-culture for monosynaptic tracing experiments. Putative DsRed$^+$GFP$^+$ starter GBM cells are indicated with an arrow. The names of the hiPSC line and patient GBOs are listed. Scale bars, 50 µm.

(B) Sample confocal images of monosynaptic rabies spread from GFP$^+$DsRed$^+$ GBM cells to adjacent cholinergic neurons. Scale bar, 50 µm. Putative DsRed$^+$GFP$^+$ starter GBM cells are indicated with an arrow.

(C) Additional sample electron micrographs of morphological contacts between presynaptic cholinergic neurons (pseudo-colored blue) and postsynaptic GBM cells (pseudo-colored red) in co-culture. Yellow stars indicate synaptic vesicles. Scale bars as indicated, either 1 µm or 500 nm.

(D) Left, uniform manifold approximation and projection (UMAP) of Neftel et al. (Neftel et al., 2019) primary GBM cells, with assignment of transcriptional cellular states (see Methods). Right, expression feature plots for the CHRM3 receptor, indicating broad expression across cellular states in primary GBM.

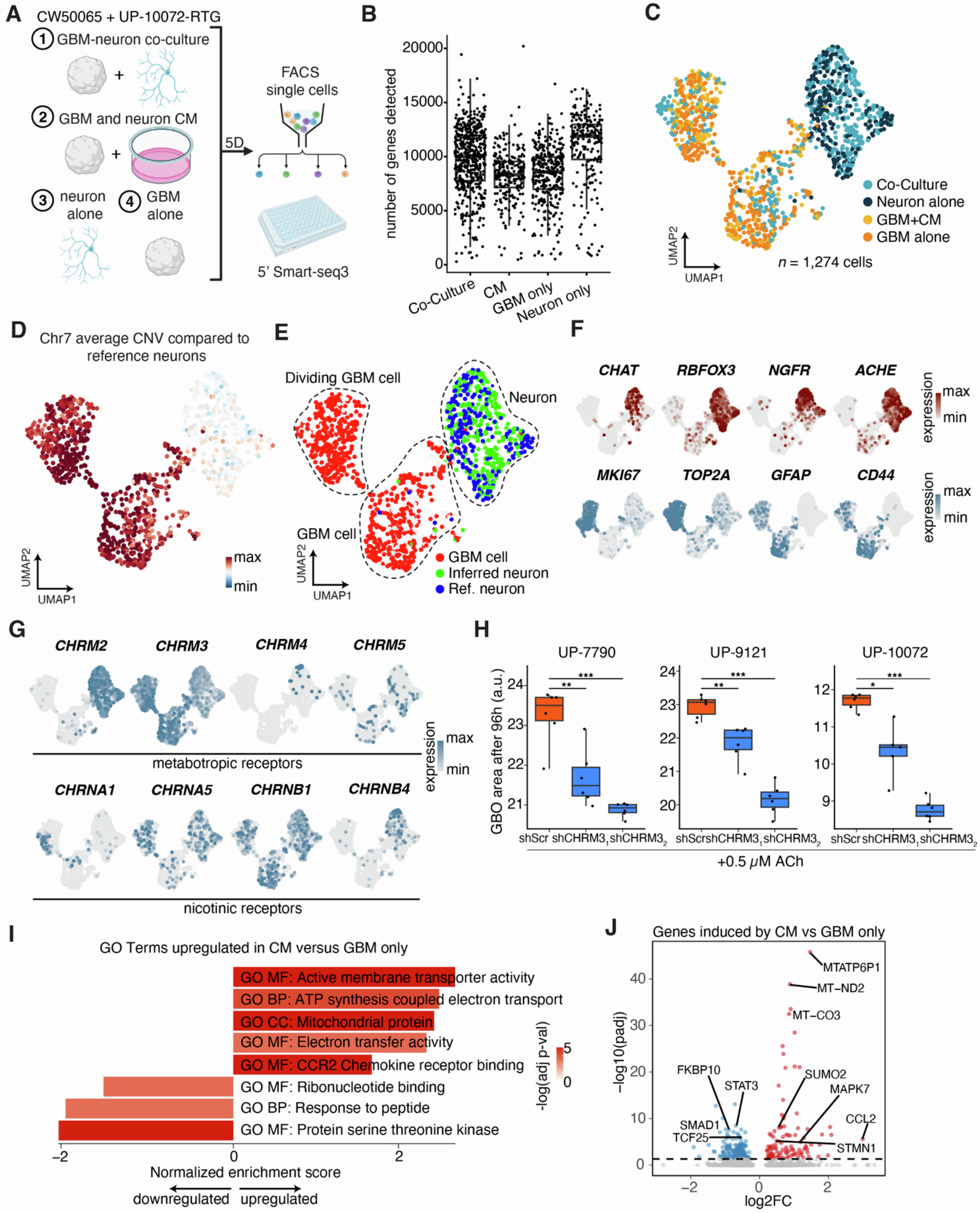

Figure S2. Single-cell transcriptional profiles of GBM cells and cholinergic neurons under different conditions, related to Figures 3 and 4.

(A) Schematic illustration of the scRNAseq experimental paradigm, consisting of either GBM-neuron co-culture, GBM cells with neuronal conditioned media (CM), neurons alone, or GBM alone cultures.
(B) Box plot of the number of genes detected from each culture condition, with each dot representing one cell.
(C-E) UMAP plots of n = 1,274 single cells colored by culture identity (C), average chromosome 7 copy number variation (CNV) (D), or by final assigned identity (E). The neuron-only culture condition (e.g., 'ref. neuron') was used as a reference to assign CNVs for the rest of the cells.
(F) Gene expression feature plots of representative cholinergic neuron genes (top) and genes expressed in malignant cells (bottom).
(G) Expression feature plots of various metabotropic and nicotinic acetylcholine receptors showing increased expression of CHRM3 across malignant cells.
(H) Boxplots of 2D measurement of areas of GBOs transduced with either a scrambled shRNA or two distinct shRNAs targeting CHRM3 cultured for 96 hours, showing decreased tumor cell viability and proliferation in the presence of 0.5 μM ACh (n = 5-6 organoids per condition; UP-7790: **p=0.0068 (shScr vs. shCHRM3$_1$), ***p=0.0006 (shScr vs. shCHRM3$_2$); UP-9121: **p=0.0046 (shScr vs. shCHRM3$_1$), ***p=1.2x10^{-6} (shScr vs. shCHRM3$_2$); UP-10072: *p=0.024 (shScr vs. shCHRM3$_1$), ***p=8.2x10^{-9} (shScr vs. shCHRM3$_2$); Welch's t-tests with Bonferroni's correction for multiple comparisons).
(I-J) Representative GO terms (I) and volcano plots (J) of genes induced by CM by comparing tumor cells in the CM condition versus GBM alone, similar to Figures 4E-4F.

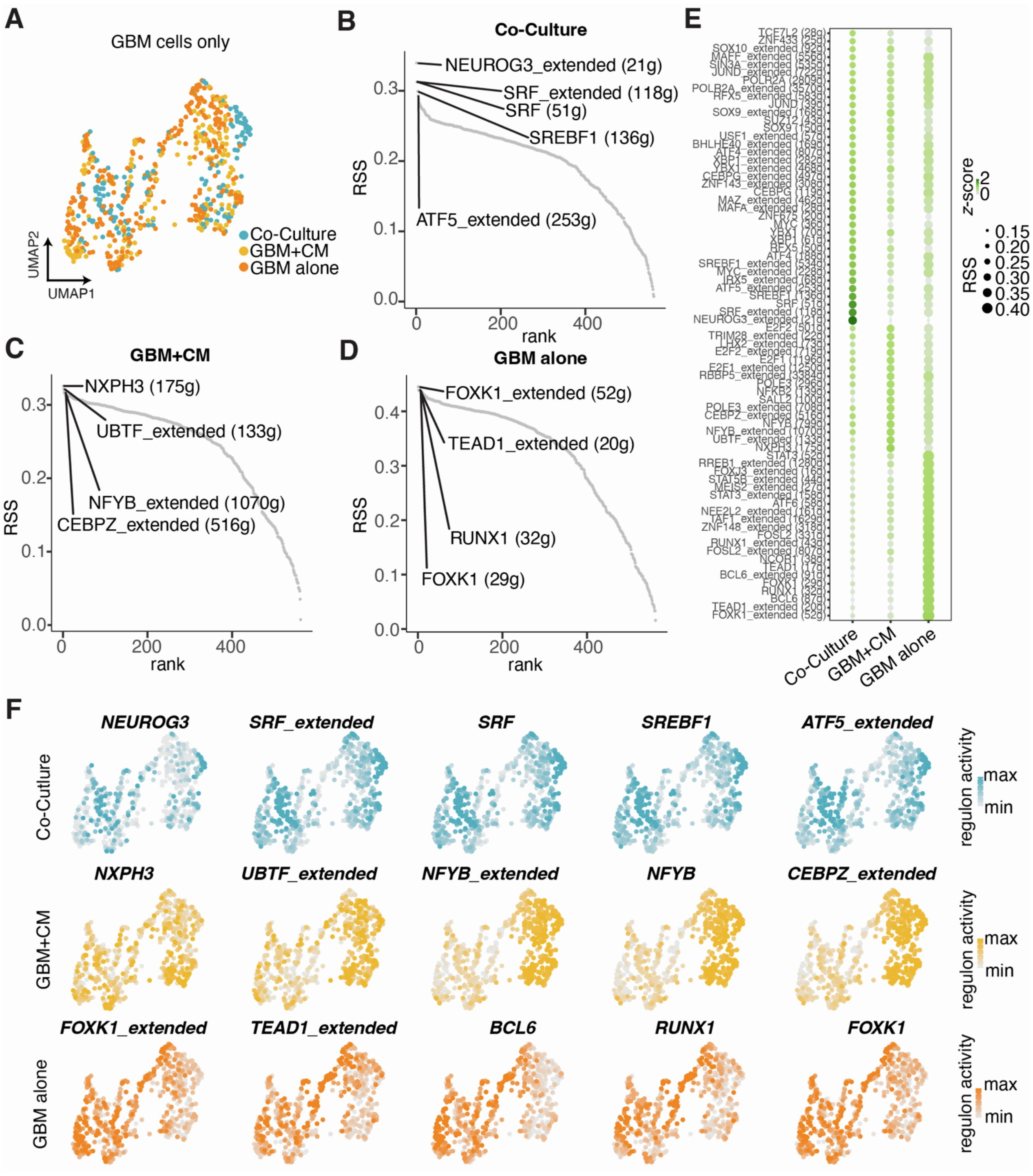

Figure S3*. *Transcriptional regulators of GBM cells in the presence of cholinergic neurons identified by gene regulatory network (GRN) analyses, related to Figures 3 and 4.

(A) UMAP plots of *n* = 672 single cells confidently assigned to malignant identity, colored by condition. CM, conditioned media.

(B-D) Regulon specificity scores in co-culture (B), CM (C), or GBM alone (D) conditions. Top motifs are indicated for each condition.

(E) Bubble plot of top regulons in each condition.

(F) Feature plots of the activity of top regulons for each condition as implemented in the SCENIC package (Aibar et al., 2017).

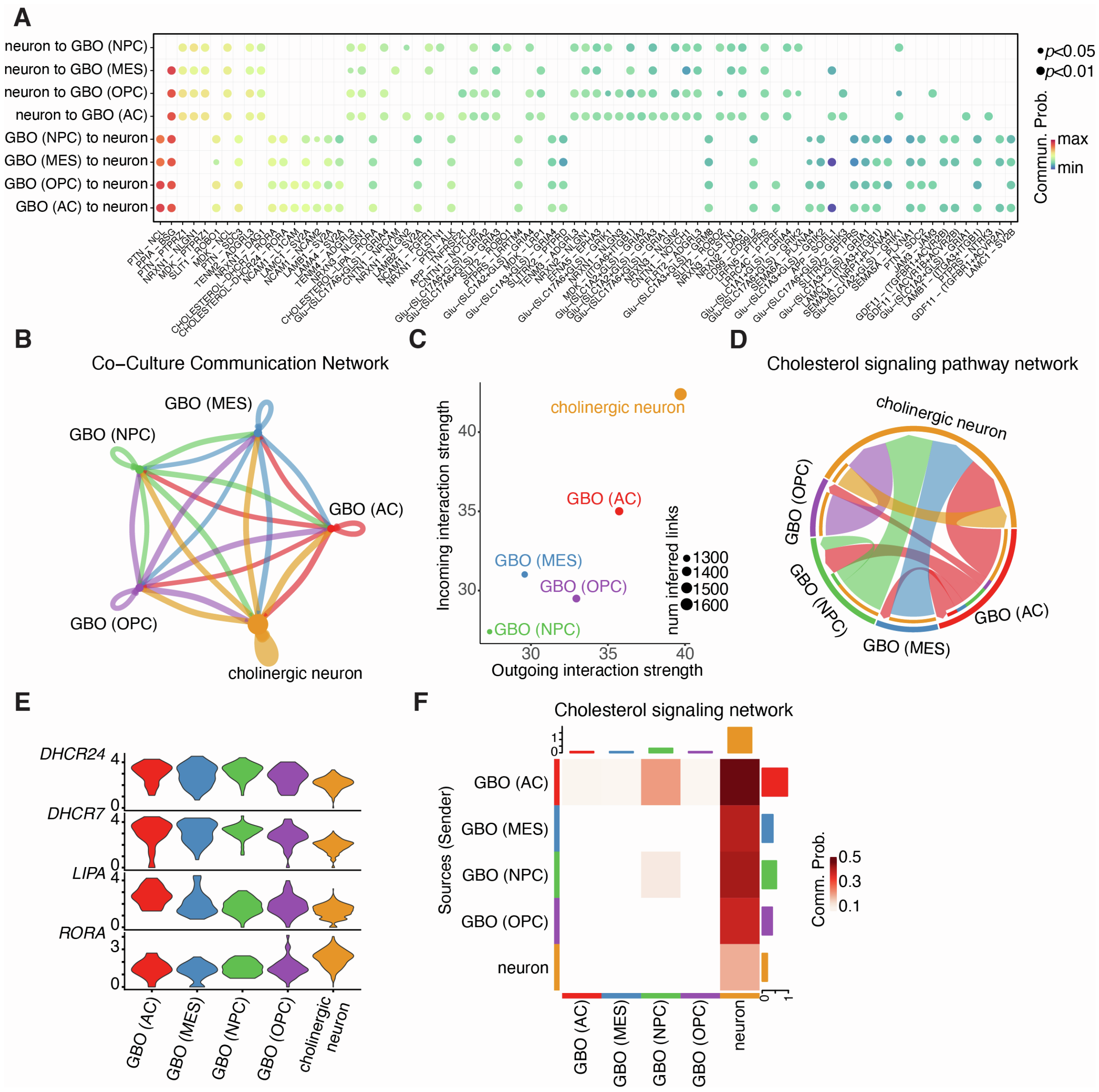

Figure S4. Major signaling pathways between cholinergic neurons and GBM cells of different states in co-culture identified by cell-cell interaction analyses, related to Figures 3 and 4.
(A) Bubble plot of significant inferred cell-cell interactions between cholinergic neurons and AC-like, OPC-like, NPC-like, or MES-like GBM cells.
(B) Circle diagram of aggregated cell-cell communication network between GBM cells of different states and neurons.
(C) Visualization of dominant senders and receivers in co-culture based on number of inferred links.
(D-F) Analyses highlighting the importance of the cholesterol metabolism pathway in GBM-cholinergic neuron interactions via a chord diagram (D), violin plots of relevant pathway genes (E), and heatmap of the cholesterol signaling network (F).

SUPPLEMENTARY TABLES (in Excel)

Table S1. GBO and hiPSC line additional information, related to Figures 1-4.
Flow-chart of experiments performed in the study and the associated GBO line(s) and/or hiPSC line(s) used to derive cholinergic neurons as applicable.

Table S2. Differentially expressed genes in GBM cells under different conditions, related to Figures 3-4.
Lists of differentially expressed genes between GBM cells in co-culture versus conditioned media, co-culture versus tumor alone, or conditioned media versus tumor alone.

Table S3. Primer sequences for shRNA targeting, related to Figure S2.
Oligonucleotide sequences for plasmid construction for knockdown of CHRM3. Sequences for CHRM3 KD 2 and Scramble were previously reported in (Sun et al., 2025).

SUPPLEMENTARY METHODS

Immunohistochemistry

For immunohistochemistry, coverslips were washed 2X with ice-cold DPBS, gently fixed for 20 minutes using ice-cold 4% paraformaldehyde (PFA), and washed 2X again with DPBS, prior to incubation with blocking buffer containing (TBS with 0.1% Tween-20 (Sigma-Aldrich, T8787-50ML), 0.5% Triton X-100, 10% donkey serum (Millipore, S30), 1% BSA (Sigma-Aldrich, B6917), and 22.52 mg/mL glycine (Sigma-Aldrich, 50046-50G) for 1 hour at room temperature (RT). Coverslips were then incubated in diluted primary antibodies in antibody buffer (TBS with 0.1% Tween-20, 0.5% Triton X-100, and 5% donkey serum) overnight at 4°C. The next day, coverslips were washed 3X in TBST (TBS with 0.1% Tween-20) for 5 minutes each and incubated with secondary antibodies diluted in antibody buffer as described above for 1-2 hours at RT. Where applicable, DAPI (Thermo Fisher Scientific, D1306, 1:500) was added during the secondary antibody incubation. Coverslips were washed 3X in TBST for 5 minutes each and then mounted on a glass slide (Thermo Fisher Scientific, 1518848) in mounting medium (Vector Laboratories, H-1000-10) and sealed with nail polish prior to confocal imaging (Zeiss LSM 810 or Zeiss LSM 710) as *z*-stacks with either 10X, 20X, or 40X objectives.

The following primary antibodies were used: Goat anti-RFP (Biorbyt, orb11618, 1:500), rabbit anti-RFP (Rockland, 600-401-379,1:500), chicken anti-GFP (Abcam, ab13970, 1:2000), goat anti-GFP (Rockland, 600-101-215, 1:500), goat anti-ChAT (Sigma-Aldrich, AB144P-200UL, 1:200), rabbit anti-VAChT (Synaptic Systems, 139103, 1:500), mouse anti-KI67 (BD Biosciences, 550609, 1:500), rabbit anti-KI67 (Abcam, ab16667, 1:500), mouse anti-NeuN (Thermo Fisher Scientific, MA5-33103, 1:500), and chicken anti-beta-microtubules 3 (Aves, TUJ-0020, 1:1000). The following secondary antibodies were used: donkey anti-chicken Alexa Fluor 488 (Thermo Fisher Scientific, A-78948, 1:500), donkey anti-mouse Alexa Fluor 555 (Thermo Fisher Scientific, A-31570, 1:500), donkey anti-goat Alexa Fluor 647 (Thermo Fisher Scientific, A-21447, 1:500), donkey anti-rabbit Alexa Fluor 555 (Thermo Fisher Scientific, A-31572, 1:500), donkey anti-mouse Alexa Fluor 647 (Thermo Fisher Scientific, A-31571, 1:500), and donkey anti-rabbit Alexa Fluor 405 (Thermo Fisher Scientific, A-48258, 1:500).

Viral vectors and plasmids

EnvA-pseudotyped G-deleted EGFP rabies virus was purchased from the Salk viral core (32635). The GBOs expressing the retroviral RTG helper plasmid (Addgene #235698) were generated as described previously (Sun et al., 2025). The UP-10072 GBOs expressing the red-shifted calcium indicator jRGECO1α were generated as described previously (Dana et al., 2016; Sun et al., 2025). The lentiviral construct for optogenetic depolarization of cholinergic neurons was generated using

the pLenti-EF1a-hChR2(H134R)-EYFP-WPRE plasmid (Addgene, 20942). shRNAs were generated as described previously (Sun et al., 2025), with targeting sequences listed in Table S3.

Calcium imaging and analyses

For Ca^{2+} imaging of co-cultures, pure cholinergic neurons (CW50065) were infected with lenti-ChR2 at 3 days *in vitro* (DIV). At 4 weeks *in vitro*, UP-10072 GBO cells expressing jRGECO1α were seeded into plates at a 1:20 tumor cell to neuron ratio for Ca^{2+} imaging. Three days after seeding, live Ca^{2+} imaging was performed using a confocal microscope (Zeiss LSM 710) with a 10X objective by acquiring images at 2 Hz in the 555 nm wavelength channel. Light pulses for optogenetic stimulation of cholinergic neurons were delivered by a laser (LRD-0470-PFFD-00100-05) with 470 nm wavelength (~0.32 mW/mm^2 power) connected to a power supply (PSU-H-LED), with pulse length and frequency set by a programmable pulse generator (Master 8). Simultaneous Ca^{2+} imaging and optogenetic stimulation (10 ms pulses at 20 Hz for 10 seconds) were performed with or without the presence of 100 μM 4-DAMP.

In brief, recordings were exported as .CZI files and imported to ImageJ/FIJI for quantification. Cells that exhibited Ca^{2+} transients in response to optogenetic stimulation were analyzed. To generate the dF/F traces, we first obtained the baseline intensity trace by computing the tenth percentile of a moving 50-frame window of each raw trace using the rollapply function in R. The dF/F trace was then defined as the $\frac{\text{raw intensity} - \text{baseline intensity}}{\text{baseline intensity}}$ at each timepoint. The trace was then smoothed twice (triangular moving average) via a 7-frame window. We defined $\Delta dF/F_{max}$ as the maximum change in dF/F between the mean dF/F in a 10-second window prior to light stimulation compared to maximal dF/F.

Post-fixation processing for electron microscopy

Coverslips then washed 3X for 5 minutes each in 0.1 M PB and postfixed 1h in 1% osmium tetroxide in 0.1 M PB. They were then washed 3X for 5 minutes each in 0.1M PB, then in double distilled water for 1 minute and dehydrated in an ascending series of ethanol (50%, 70%, 90%, 100%), 7 minutes each, followed by acetone 100% 3X for 7 minutes each. Cells were then cured in epoxy resin:acetone mix (1:1; 2:1) for 30 minutes each, and epoxy 100% for 10 minutes. Coverslips were removed from the holders and placed face up on a glass slide and cured for 1h at 50°C. After this, a plastic capsule filled with resin was placed on top of each coverslip and cured for 48h at 60°C. The coverslips were removed from the blocks by plunging into liquid nitrogen. Blocks were then trimmed, and serial sections (50nm thickness) were collected on a single-slot formvar-coated copper grid. Sections were contrasted with uranyl acetate and lead citrate and observed at the

electron microscopy facility of the University of Lausanne, on a 120KV Talos transmission electron microscope at a magnification of 8500x.

scRNAseq data processing and analysis

Single cell RNA sequencing data were processed as previously described (Sun et al., 2025) prior to analysis. Count matrices generated using the “GeneFull” option in STARsolo, which includes intronic counts, were imported into R (v4.3.1) using the Seurat package (v4.3.0.1). Cells containing fewer than 1000 UMIs or more than 20% mitochondrial UMIs were excluded. Data normalization was performed using SCTransform with vst.flavor = "v2", variable.features.n = 15000, and regression on mitochondrial UMIs percentage and total UMI counts. Copy number aberration (CNA) analysis was conducted using the HoneyBADGER R package (Fan et al., 2018). GBM cellular states were assigned as previously described (Neftel et al., 2019), utilizing the get.sig.scores function (LeBlanc et al., 2022). Primary patient GBM transcriptional data (Neftel et al., 2019) were integrated with harmony (Korsunsky et al., 2019). Differentially expressed genes (DEGs) were identified using Seurat’s FindAllMarkers function, applying a log fold-change threshold of 0.1 and an adjusted p-value < 0.05 (Wilcoxon rank-sum test with Bonferroni correction). Gene Ontology (GO) analyses were performed using fgsea package in R (Korotkevich et al., 2021). Gene regulatory network analyses were conducted with SCENIC in R (Aibar et al., 2017) and cell-cell interaction analyses were performed with CellChat v2 (Jin et al., 2025) with default parameters.

Additional references

Aibar, S., González-Blas, C.B., Moerman, T., Huynh-Thu, V.A., Imrichova, H., Hulselmans, G., Rambow, F., Marine, J.-C., Geurts, P., Aerts, J., et al. (2017). SCENIC: single-cell regulatory network inference and clustering. Nat Methods *14*, 1083–1086. https://doi.org/10.1038/nmeth.4463.

Dana, H., Mohar, B., Sun, Y., Narayan, S., Gordus, A., Hasseman, J.P., Tsegaye, G., Holt, G.T., Hu, A., Walpita, D., et al. (2016). Sensitive red protein calcium indicators for imaging neural activity. eLife *5*, e12727. https://doi.org/10.7554/eLife.12727.

Fan, J., Lee, H.-O., Lee, S., Ryu, D., Lee, S., Xue, C., Kim, S.J., Kim, K., Barkas, N., Park, P.J., et al. (2018). Linking transcriptional and genetic tumor heterogeneity through allele analysis of single-cell RNA-seq data. Genome Res. gr.228080.117. https://doi.org/10.1101/gr.228080.117.

Jin, S., Plikus, M.V., and Nie, Q. (2025). CellChat for systematic analysis of cell–cell communication from single-cell transcriptomics. Nat Protoc *20*, 180–219. https://doi.org/10.1038/s41596-024-01045-4.

Korotkevich, G., Sukhov, V., Budin, N., Shpak, B., Artyomov, M.N., and Sergushichev, A. (2021). Fast gene set enrichment analysis. 060012. https://doi.org/10.1101/060012.

Korsunsky, I., Millard, N., Fan, J., Slowikowski, K., Zhang, F., Wei, K., Baglaenko, Y., Brenner, M., Loh, P., and Raychaudhuri, S. (2019). Fast, sensitive and accurate integration of single-cell data with Harmony. Nat Methods *16*, 1289–1296. https://doi.org/10.1038/s41592-019-0619-0.

LeBlanc, V.G., Trinh, D.L., Aslanpour, S., Hughes, M., Livingstone, D., Jin, D., Ahn, B.Y., Blough, M.D., Cairncross, J.G., Chan, J.A., et al. (2022). Single-cell landscapes of primary glioblastomas and matched explants and cell lines show variable retention of inter- and intratumor heterogeneity. Cancer Cell *40*, 379-392.e9. https://doi.org/10.1016/j.ccell.2022.02.016.

Neftel, C., Laffy, J., Filbin, M.G., Hara, T., Shore, M.E., Rahme, G.J., Richman, A.R., Silverbush, D., Shaw, M.L., Hebert, C.M., et al. (2019). An Integrative Model of Cellular States, Plasticity, and Genetics for Glioblastoma. Cell *178*, 835-849.e21. https://doi.org/10.1016/j.cell.2019.06.024.

Sun, Y., Wang, X., Zhang, D.Y., Zhang, Z., Bhattarai, J.P., Wang, Y., Park, K.H., Dong, W., Hung, Y.-F., Yang, Q., et al. (2025). Brain-wide neuronal circuit connectome of human glioblastoma. Nature 1–3. https://doi.org/10.1038/s41586-025-08634-7.
