# Supplementary material for: Cholinergic neuron-to-glioblastoma synapses in a human iPSC-derived co-culture model
Source: Stem Cell Reports. 2025 Jun 19;20(7):102534. doi: 10.1016/j.stemcr.2025.102534 (PMC12277791; doi:10.1016/j.stemcr.2025.102534)
Supplement: Document S2. Article plus supplemental information [file mmc5.pdf]

Stem Cell Reports
Report
OPEN ACCESS

Cholinergic neuron-to-glioblastoma synapses in a human iPSC-derived co-culture model

Yusha Sun,[1,11] Xin Wang,[2,11] Zhijian Zhang,[2,11] Kristen H. Park,[1] Yicheng Wu,[2] Weifan Dong,[2] Daniel Y. Zhang,[3] Yao Fu,[2] Feng Zhang,[2] Zev A. Binder,[3,4] Emily Ling-Lin Pai,[5] MacLean P. Nasrallah,[4,5] Kimberly M. Christian,[2] Donald M. O'Rourke,[3,4] Nicolas Toni,[6] Guo-li Ming,[2,7,8,9,*] and Hongjun Song[2,3,4,8,10,12,*]

[1]Neuroscience Graduate Group, Perelman School of Medicine, University of Pennsylvania, Philadelphia, PA, USA
[2]Department of Neuroscience and Mahoney Institute for Neurosciences, Perelman School of Medicine, University of Pennsylvania, Philadelphia, PA, USA
[3]Department of Neurosurgery, Perelman School of Medicine, University of Pennsylvania, Philadelphia, PA, USA
[4]Glioblastoma Translational Center of Excellence, The Abramson Cancer Center, Perelman School of Medicine, University of Pennsylvania, Philadelphia, PA, USA
[5]Department of Pathology and Laboratory Medicine, Perelman School of Medicine, University of Pennsylvania, Philadelphia, PA, USA
[6]Center for Psychiatric Neurosciences, Lausanne University Hospital, University of Lausanne, Lausanne, Switzerland
[7]Institute for Regenerative Medicine, University of Pennsylvania, Philadelphia, PA, USA
[8]Department of Cell and Developmental Biology, Perelman School of Medicine, University of Pennsylvania, Philadelphia, PA, USA
[9]Department of Psychiatry, Perelman School of Medicine, University of Pennsylvania, Philadelphia, PA, USA
[10]The Epigenetics Institute, Perelman School of Medicine, University of Pennsylvania, Philadelphia, PA, USA
[11]These authors contributed equally
[12]Lead contact
*Correspondence: gming@pennmedicine.upenn.edu (G.-l.M.), shongjun@pennmedicine.upenn.edu (H.S.)
https://doi.org/10.1016/j.stemcr.2025.102534

SUMMARY

Glioblastoma (GBM) integrates extensively into brain-wide neuronal circuits; however, neuron-tumor interactions have largely been studied with glutamatergic neurons in animal models. The role of neuromodulatory circuits for GBM biology in all-human cell systems remains unclear. Here, we report a co-culture system employing patient-derived GBM organoids and human induced pluripotent stem cell (hiPSC)-derived cholinergic neurons. We provided evidence of structural human cholinergic synaptic inputs onto GBM cells via trans-monosynaptic tracing and electron microscopy and functional synaptic interactions through the metabotropic CHRM3 receptor via calcium imaging. Deep single-cell RNA sequencing of co-cultures compared to GBM monocultures further revealed shifts in tumor transcriptional profiles toward a more proliferative state, with contributions from both diffusible factors and direct contacts, the latter of which are dependent on cholesterol biosynthesis. Together, our findings support the role of cholinergic inputs in promoting GBM progression and highlight hiPSC-derived co-culture models as a useful platform for cancer neuroscience.

INTRODUCTION

The role of neuronal influences on cancer pathogenesis and progression is increasingly appreciated in the nervous system (Monje et al., 2020). Neurons enhance glioma proliferation and migration via diffusible paracrine factors or synaptic inputs onto tumor cells (Monje et al., 2020). In glioblastoma (GBM), mostly glutamatergic inputs have been identified (Venkataramani et al., 2019; Venkatesh et al., 2019). While the potential for GBM to receive projections from neurons of other neurotransmitter subtypes, such as from cholinergic neurons, has recently been discovered in xenotransplantation models (Hsieh et al., 2024; Sun et al., 2025; Tetzlaff et al., 2024), the impact of these diverse subtypes on tumor biology in all-human cell-based systems is still unclear.

We previously introduced transsynaptic viral tracing tools to define monosynaptically projecting neurons to GBM cells in mice and found that basal forebrain cholinergic neurons can interact with GBM (Sun et al., 2025). However, whether synapses can form between human cholinergic neurons and GBM cells and consequences of these inputs and other non-synaptic mechanisms on GBM cells are still unknown. Human induced pluripotent stem cell (hiPSC)-based models have been emerging as a powerful platform for studying human-specific disease mechanisms (Zhou et al., 2024). In this study, we developed a co-culture model for the study of neuron-tumor interactions by combining patient-derived glioblastoma organoids (GBOs) (Jacob et al., 2020) and hiPSC-derived cholinergic neurons. We provided evidence for direct human cholinergic synaptic inputs onto GBM cells. We further showed that human cholinergic neurons can drive tumor cell transcriptional reprogramming to promote tumor fitness via both contact-dependent and -independent means, including via an upregulation of cholesterol metabolism, which could be a targetable dependency in GBM.

Published by Elsevier Inc. on behalf of International Society for Stem Cell Research.
This is an open access article under the CC BY license (http://creativecommons.org/licenses/by/4.0/).

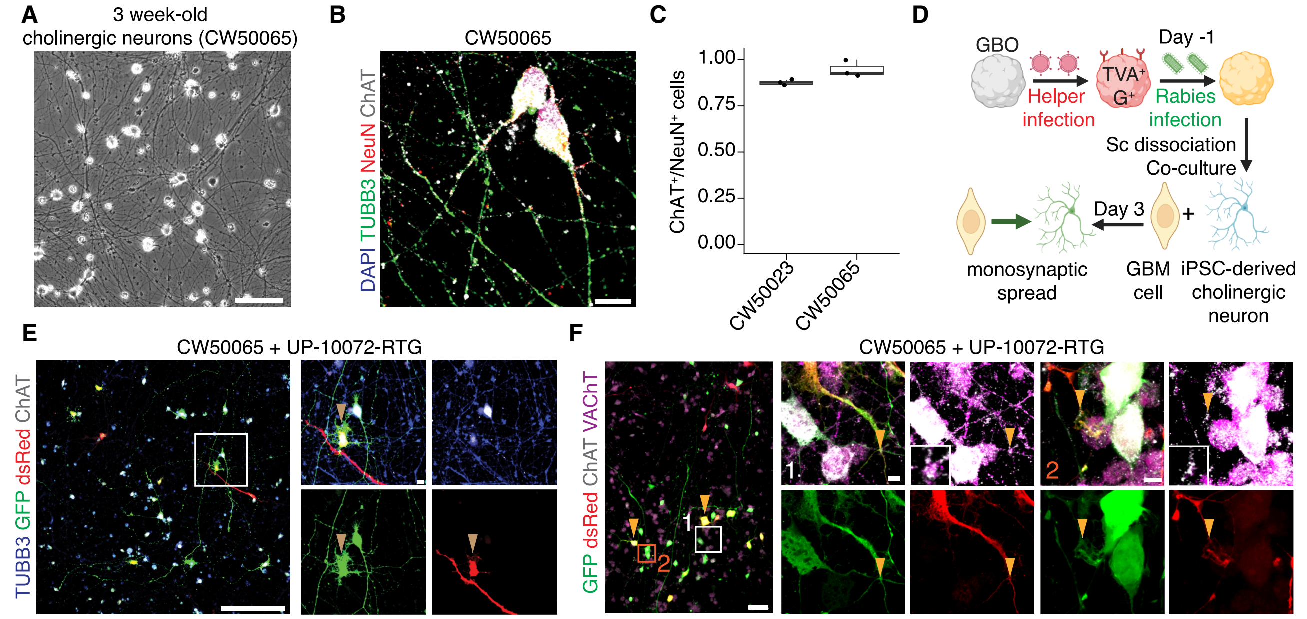

Figure 1. Trans-monosynaptic tracing in human cholinergic neuron-GBM co-cultures

(A) Sample bright-field image of hiPSC-derived cholinergic neurons after 3 weeks *in vitro*. Scale bar, 200 μm.

(B) Sample confocal images of cholinergic neuron cultures. Scale bar, 10 μm.

(C) Quantification of the proportion of NeuN$^+$ neurons that were ChAT$^+$ (quantified from $n = 3$ biological replicates, representative of $n = 2$ distinct differentiations from $n = 2$ hiPSC lines; 42 neurons for CW50065 and 47 neurons for CW50023 were counted).

(D) Schematic illustration of rabies virus-based trans-monosynaptic tracing paradigm in cholinergic neuron-GBM co-cultures, leading to viral spread by 3 days.

(E) Sample confocal images of trans-monosynaptic rabies virus spread from DsRed$^+$GFP$^+$ starter GBM cells (arrow) to adjacent ChAT$^+$ neurons. Scale bars, 200 and 10 μm (insets).

(F) Sample confocal images revealing sites of close contact with dense ChAT$^+$VAChT$^+$ puncta. Arrows in enlarged image on the left denote DsRed$^+$GFP$^+$ GBM starter cells, and arrows in the insets on the right denote neuron-glioma contacts that could represent putative sites of rabies virus spread. Scale bars, 50 and 5 μm (insets). See also Figure S1 and Table S1.

RESULTS

Structural cholinergic neuron-to-GBM synapses in an all-human cell model

We first assessed whether structural synapses form between human cholinergic neurons and human GBM cells. We leveraged a commercial source of cholinergic neurons derived from hiPSCs of two donors using a transcription factor-based approach (Table S1). Cholinergic neurons were highly pure by 3 weeks *in vitro* (Figures 1A–1C). We used GBM cells dissociated from GBOs derived from three patients for co-culture (Table S1) (Jacob et al., 2020). We then performed monosynaptic rabies virus tracing between GBM cells and human ChAT$^+$ neurons to assess the potential for synapse formation (Figures 1D–1F). GBOs were transduced with a rabies helper vector and were pre-infected with rabies virus before dissociation and co-culture with cholinergic neurons (Figure 1D) (Sun et al., 2025). After 2–3 days in co-culture, we observed GFP$^+$DsRed$^-$ neurons adjacent to starter GBM cells using hiPSC lines from two donors and GBOs from three patients, suggesting rapid trans-monosynaptic spread of rabies virus from postsynaptic tumor cells to presynaptic cholinergic neurons (Figures 1E, S1A, and S1B). We additionally observed dense ChAT$^+$VAChT$^+$ puncta at sites of neuron-tumor contacts, supporting the existence of synaptic contacts (Figure 1F).

Next, we examined the ultrastructure of these tumor-neuron co-cultures with transmission electron microscopy (EM). Under EM, clear multi-nucleated GBM cells exhibited disorganized cytoskeletal structures (Figures 2A–2C and S1C). We found axons with clear vesicles in close juxtaposition to tumor cells, consistent with a direct synaptic connection (Figures 2A, 2B, and S1C). Collectively, our data provide structural evidence for the existence of a cholinergic neuron-to-GBM synaptic connection in all human cell systems *ex vivo*.

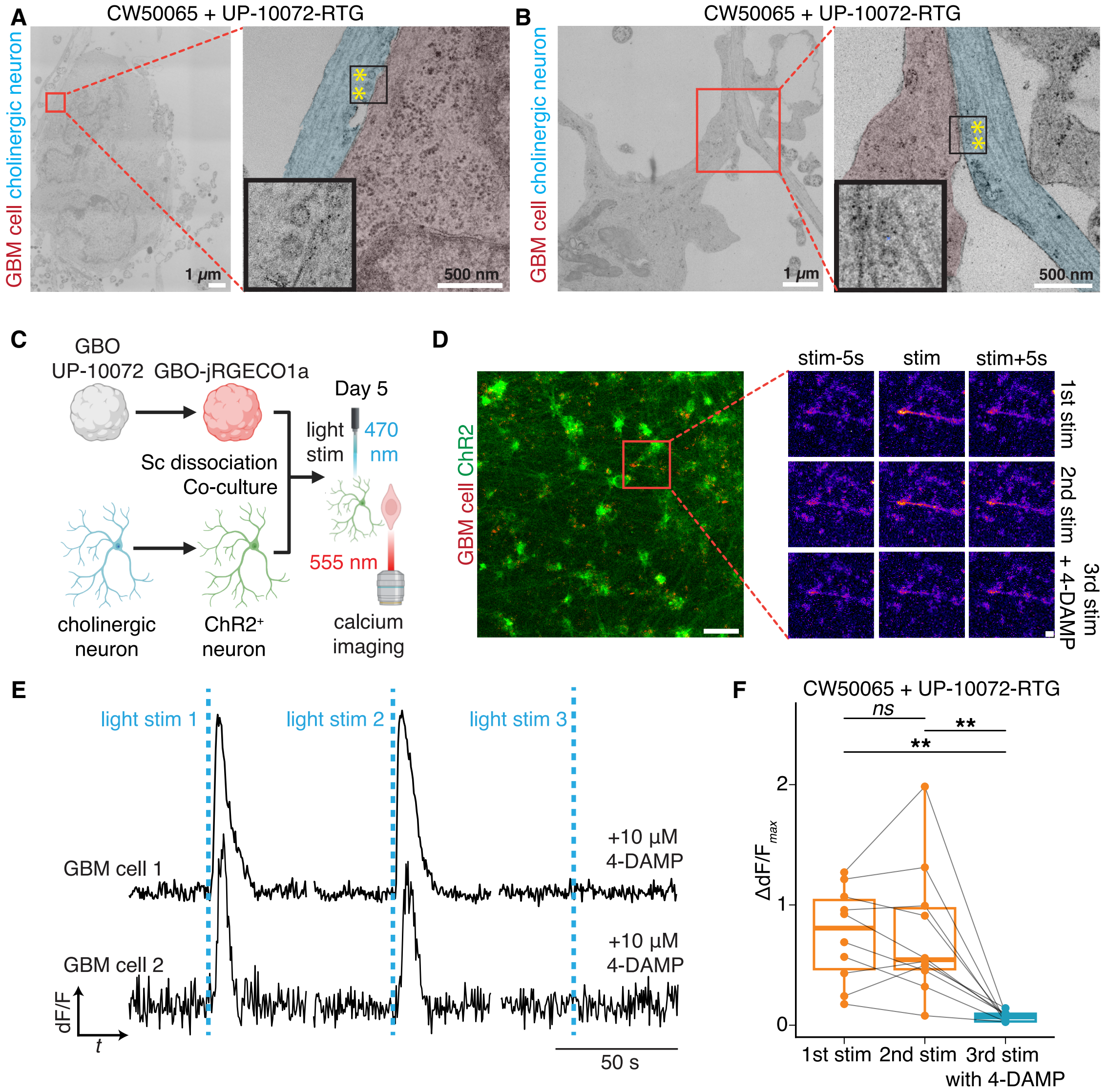

Figure 2. Structural and functional evidence for human cholinergic neuron-to-GBM synapses mediated by CHRM3

(A and B) Sample electron micrographs of morphological contacts between presynaptic cholinergic neurons (pseudo-colored blue) and postsynaptic GBM cells (pseudo-colored red) in co-culture. Yellow stars indicate synaptic vesicles. Scale bars: 1 or 500 nm.

(C) Schematic illustration of Ca^{2+} imaging paradigm with co-cultures, with simultaneous light stimulation of cholinergic neurons (at 470 nm wavelength) and Ca^{2+} imaging of GBOs (at 555 nm wavelength).

(D) Sample confocal images from Ca^{2+} imaging, showing responses of a cell at first stimulation, second stimulation, and third stimulation after the CHRM3 blockade by 4-DAMP. Scale bars, 100 and 20 μm (insets).

(E and F) Sample traces (E) and quantification (F) of fluorescence intensity traces of GBM cells after light stimulations. Quantifications are of the maximum Ca^{2+} response in response to light stimulation relative to baseline ($n = 10$ individual cells from $n = 3$ biological replicates); 1st stim. vs. 2nd stim.: $p = 0.92$; 1st stim. vs. 3rd stim.: $***p = 0.001$; 2nd stim. vs. 3rd stim.: $**p = 0.005$; paired Welch's t tests with false discovery rate (FDR) adjustment for multiple comparisons. See also Figure S1 and Tables S1 and S3.

Functional evidence for human cholinergic neuron-GBM synapses in co-culture

We next examined whether human cholinergic neurons could functionally modulate GBM cells via Ca^{2+} imaging. We transduced cholinergic neurons at 3 days *in vitro* with a lentivirus expressing ChR2 and co-cultured them with GBM cells expressing the red-shifted calcium indicator jRGECO1α (Sun et al., 2025) 18 days later (Figure 2C). Five days after co-culture, we performed simultaneous optogenetic stimulation and Ca^{2+} imaging of tumor-neuron

cultures (Figure 2C). GBM cells responded immediately to optogenetic stimulation of cholinergic neurons across two consecutive trials, consistent with a synaptic response (Figures 2C–2F). After the addition of 4-DAMP (1,1-dimethyl-4-diphenylacetoxypiperidinium iodide), a blocker specific for the metabotropic CHRM3 receptor (Thomas et al., 1992), the GBM response to light stimuli was completely abrogated (Figures 2C–2F). These findings are consistent with the high expression of CHRM3 observed in published single-cell RNA sequencing (scRNA-seq) data from primary GBM (Figure S1D) (Neftel et al., 2019).

Together, our findings provide evidence for a functional cholinergic neuron-to-GBM synapse between cells of human origin in co-culture and raise the possibility that these inputs may have functional implications for GBM biology.

Human cholinergic neurons drive neural and proliferative programs in GBM cells

We next asked how cholinergic neurons may affect tumor cells transcriptionally. We co-cultured GBM cells with cholinergic neurons or cultured either GBM cells or cholinergic neurons alone, all in the same medium, and performed scRNA-seq at 6 days post co-culture (Figure S2A). To more specifically dissect the influence of direct neuron-to-GBM interactions, including synaptic interactions, versus the effect of secreted diffusible factors, we also profiled GBM cells cultured in the conditioned media of pure cholinergic neurons (Figures S2A and S2B). We annotated cell types by leveraging the neuron-only cells to infer copy-number aberrations (CNAs), which resulted in three distinct populations comprising cholinergic neurons, GBM cells, and dividing GBM cells (Figures S2C–S2F).

Next, we compared the transcriptomic differences between these conditions. Assignment of GBM cell states (Neftel et al., 2019) revealed a shift toward neural progenitor cell (NPC)-like and oligodendrocyte progenitor cell (OPC)-like states in both co-culture and conditioned medium conditions compared to GBM only, with GBM cells in co-culture attaining nearly 50% NPC-like/OPC-like cells compared to ~25% at the baseline (Figure 3A). Compared to the GBM-only condition, GBM cells in co-culture upregulated neuronal genes (e.g., *NEFL*, *STMN2*, and *NEUROG3*), lipid metabolism genes (e.g., *INSIG1*, *LDLR*, and *CDHR1*), and cell growth pathways such as E2F targets and mTORC1 signaling (Figures 3B and 3C).

Functionally, we found that co-culture with cholinergic neurons increased the proliferation rate of GBM cells compared to GBM culture alone (Figures 3D and 3E). Live imaging also showed an increased rate of directed cell motility, as measured by the displacement of cells from their initial position, for GBM cells in co-culture compared to culture alone (Figure 3F).

Analysis of expression levels of acetylcholine receptors revealed CHRM3 as the most widespread and robustly expressed receptor in GBM cells (Figure S2G), consistent with the blockade of calcium responses in GBM cells by 4-DAMP (Figures 2C–2F). Accordingly, knockdown of CHRM3 in GBOs from three patients with two distinct short hairpin RNAs (shRNAs) in the presence of acetylcholine decreased GBO size by 96 h post-transduction compared to a scrambled control (Figure S2H), supporting the role of CHRM3 as a therapeutic target for GBM.

To further dissect relative contributions of diffusible factors versus direct interactions, we examined genes upregulated in GBM cells in co-culture compared to those in neuron conditioned media, which similarly revealed increased expression of lipid metabolism-related and proliferation-related pathways (e.g., MTORC1 signaling) and a downregulation of immune pathways (Figures 4A–4D; Table S2). On the other hand, genes upregulated in GBM cells in conditioned media compared to the GBM-only condition were also associated with proliferation (Figures S2I and S2J; Table S2). These data indicate that, while some transcriptional programs, such as proliferation and neuronal characteristics, exhibit graded increases in enrichment from GBM cells alone in comparison to GBM cells with neuron conditioned media to GBM cells in co-culture, other programs such as lipid metabolism are uniquely induced by direct interactions between GBM cells and cholinergic neurons.

We also performed gene regulatory network (GRN) inference with GBM cells of different conditions (Figure S3), which nominated neurogenic factor *NEUROG3*, cholesterol homeostatic regulator *SREBF1*, and stress response factor *ATF5* as putative drivers of GBM cells in co-culture (Figures S3B and S3E). These GRNs were largely distinct from GBM cells with conditioned media, which were driven by the *NXPH3* regulon, or GBM cells alone, which were driven by the *FOXK1* regulon (Figures S3C–S3E). We then performed cell-cell interaction inference analyses between GBM cells and neurons in the co-culture condition (Figure S4). We conducted ligand-receptor analyses between cholinergic neurons and GBM cells assigned as AC-like (astrocyte-like), NPC-like, OPC-like, or MES-like (mesenchymal-like) (Figures S4A and S4B). While patterns of predicted neuron-GBM cell interactions were largely consistent between tumor cells of distinct transcriptional states, including interactions related to cholesterol metabolism (Figures S4C–S4F), we identified several state-specific interactions with neurons (Figure S4A).

Finally, we functionally assessed the contribution of cholesterol biosynthesis to neuron-induced GBM proliferation. While cholesterol metabolism has been implicated as a potential therapeutic target in GBM (Fuentes-Fayos et al., 2023; Nguyen et al., 2023), these pathways have not been

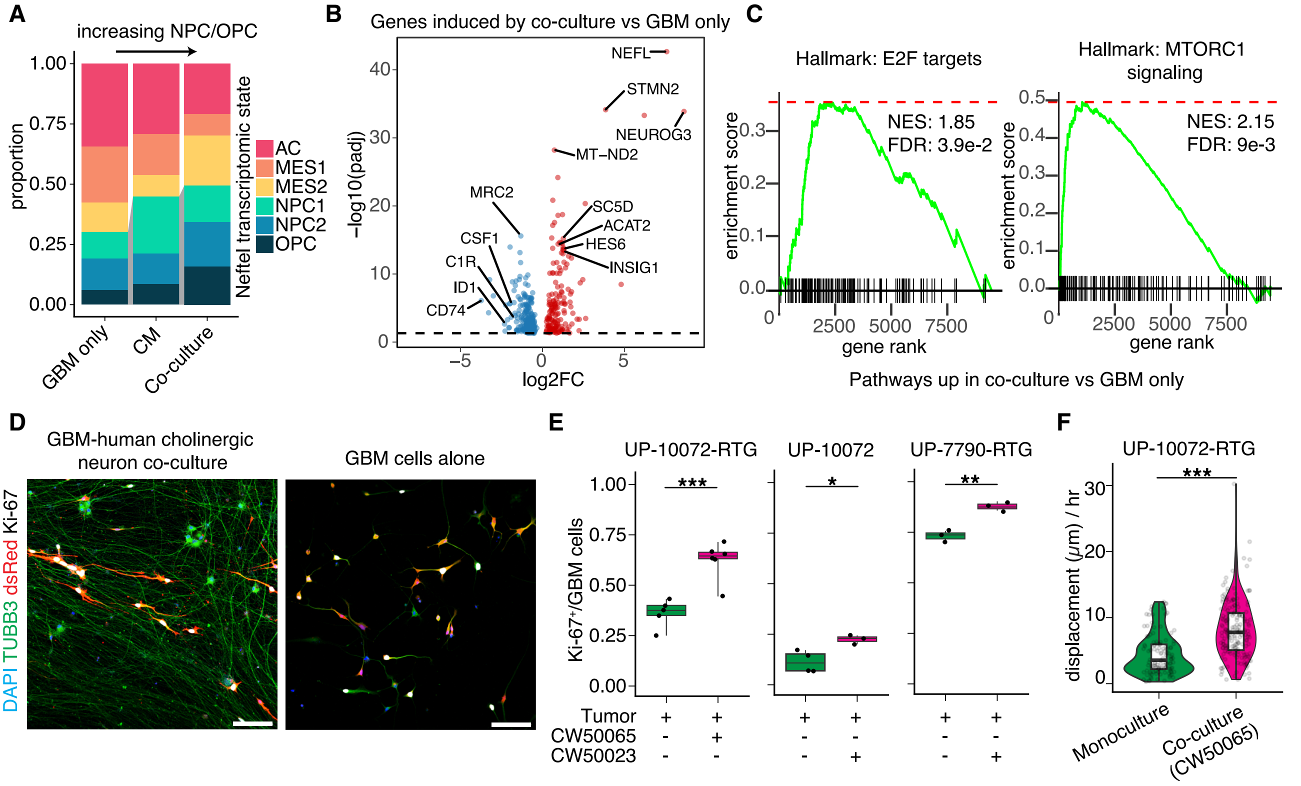

Figure 3. Human cholinergic neurons induce neural state shifts and increased proliferation in GBM cells

(A) Plot of the proportion of GBM transcriptional cell states from malignant cells in different culture conditions from scRNA-seq data (UP-10072-RTG and CW50065).

(B) Volcano plot of differentially expressed genes in GBM cells induced by co-culture versus GBM cells only.

(C) Gene set enrichment analyses (GSEA) of differentially expressed genes showing cell-cycle-related hallmark signatures, including E2F targets and MTORC1 signaling. NES, normalized enrichment score.

(D) Sample confocal images of GBM-cholinergic neuron co-cultures versus GBM cell monocultures. Scale bars, 100 μm.

(E) Quantification of the proportion of GBM cells in culture that were Ki-67$^+$ for monoculture versus co-culture conditions for two hiPSC lines and three different GBOs (UP-10072-RTG: ***$p = 0.0005$, Welch's t test; monoculture, $n = 5$ coverslips; co-culture, $n = 6$ coverslips; UP-10072: *$p = 0.018$, Welch's t test; monoculture, $n = 4$ coverslips; co-culture, $n = 3$ coverslips; UP-7790-RTG: **$p = 0.0031$, Welch's t test; monoculture, $n = 3$ coverslips; co-culture, $n = 3$ coverslips).

(F) Quantification of the displacement of GBM cells over 16 h of live imaging (***$p = 4.7 \times 10^{-15}$, Wilcoxon test; control, $n = 188$ cells from $n = 4$ coverslips; co-culture, $n = 180$ cells from $n = 6$ coverslips). See also Figures S2–S4 and Tables S1 and S2.

examined in the context of tumor-neuron interactions. Given both HMG-CoA reductase and DHCR24 were among the top genes induced by co-culture (Table S2), we performed co-culture experiments with the addition of either 5 μM simvastatin, an inhibitor of HMG-CoA reductase, or 10 μM SH-42, a selective inhibitor of DHCR24. Simvastatin appeared to abrogate the proliferative effect of cholinergic neurons on GBM cells from two patients, while SH-42 also inhibited neuron-induced proliferation of GBM cells for one patient (Figures 4E and 4F), supporting the role of cholesterol biosynthesis pathways in mediating the proliferative effects of cholinergic neurons on GBM cells.

Taken together, our findings support transcriptomic modulation of GBM via interactions with cholinergic neurons, including increased proliferative capacity via increased cholesterol biosynthesis.

DISCUSSION

It is increasingly recognized that gliomas are affected by the neural circuitry of the brain via synaptic connections and paracrine interactions (Monje et al., 2020). However, most of these prior studies focus on glutamatergic neurons, and almost all these studies were conducted in co-culture with mouse neurons or in the mouse brain. Here, we developed an all-human cell co-culture model of hiPSC-derived cholinergic neurons with primary

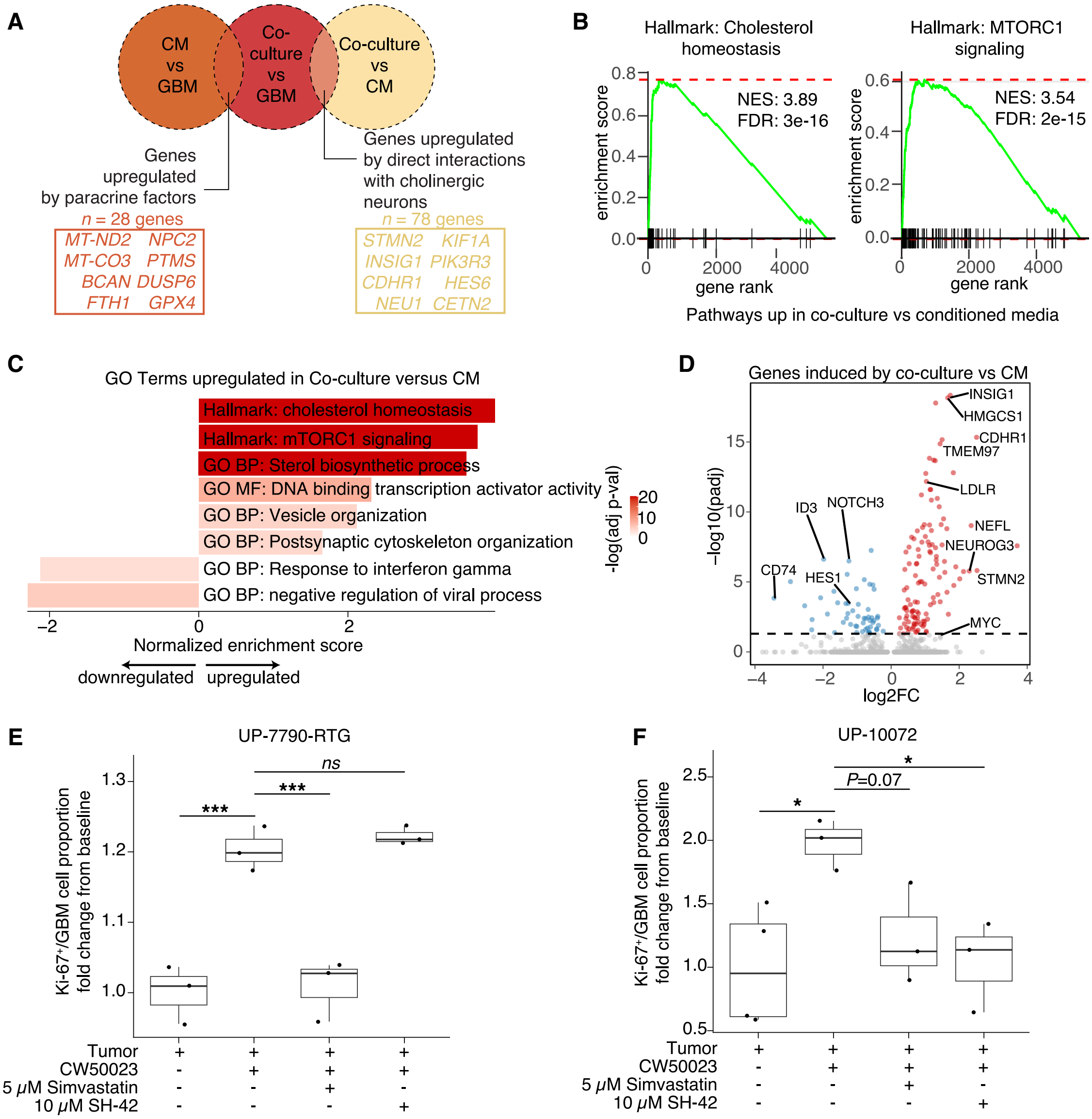

Figure 4. Transcriptional modulation of GBM by human cholinergic neurons via physical interactions and diffusible factors
(A) Venn diagram of differentially expressed genes to identify genes specifically upregulated by neuronal paracrine factors versus genes upregulated by direct interactions with cholinergic neurons (UP-10072-RTG and CW50065). Exemplary genes are listed for each condition.
(B–D) GSEA plots (B), representative GO terms (C), and volcano plots (D) of genes induced in GBM cells due to direct interactions by comparing tumor cells in the co-culture condition versus conditioned media (CM).
(E–F) Quantification of the proportion of GBM cells in culture that were Ki-67^{+} in distinct conditions with or without the presence of inhibitors of the cholesterol biosynthesis pathways for either UP-7790-RTG (E) or UP-10072 (F). Baseline data are the same as from Figure 3E. Data are plotted as a fold change from baseline Ki-67 proportion ($n = 3$ coverslips for all conditions aside from UP-10072 tumor, with $n = 4$ coverslips; UP-7790-RTG: tumor versus co-culture, ***$p = 0.00018$; co-culture versus simvastatin, ***$p = 0.00018$; co-culture versus SH-42, $p = 0.626$; UP-10072: tumor versus co-culture, *$p = 0.044$; co-culture versus simvastatin, $p = 0.07$; co-culture versus SH-42, *$p = 0.044$; pairwise comparisons with t tests with pooled SD, with p value adjustment by false discovery rate [FDR] correction). See also Figures S2–S4 and Tables S1 and S2.

patient-derived GBM cells (Wang et al., 2023). We showed structural and functional synapses between human cholinergic neurons and GBM cells, consistent with our previous findings in mouse models (Sun et al., 2025). By employing EM analyses of co-cultures, we identified pre-synaptic cholinergic neuronal axons in direct contact with postsynaptic GBM cells. As these were pure co-cultures, additional immuno-gold or other modifications to

identify cells were not necessary, thereby better preserving synaptic structures. Malignant tumor cells were unambiguously identified by the presence of multiple nuclei and highly disorganized microtubules, in contrast to neuronal axons with organized microtubules and presence of clear synaptic vesicles.

Distinct from our previous mouse transplantation study (Sun et al., 2025), in which we have examined the influence of acetylcholine on GBM migration, our current analysis of human cholinergic regulation of GBM cells revealed that neurons shift tumor transcriptional profiles toward more neural-like and proliferative states, with contributions from both diffusible factors and direct contacts. We analyzed pathways specifically induced by co-culture versus cholinergic neuron conditioned media in GBM cells and found that cholesterol and lipid metabolism were highly upregulated in co-culture. Functionally, inhibition of HMG-CoA reductase or DHCR24 largely diminished the pro-proliferative effects of direct co-culture, suggesting cholesterol biosynthesis as a mechanism by which cholinergic neurons influence GBM cells. Whether this phenomenon is unique to cholinergic neurons remains a question for further exploration. Along with recent reports evaluating the role of lipid metabolism in promoting progression of glioma and in other diseases (Clayton et al., 2024; Zhao et al., 2024), our findings suggest the potential importance of these pathways in neuron-tumor interactions and define potential targets for future preclinical studies.

The advancement of protocols enabling the differentiation of iPSCs into highly pure subtype-specific neurons in 2D cultures and brain region-specific 3D organoids provides versatile platforms for studying neuron-tumor interactions in the form of co-culture or assembloids (Krieger et al., 2020; Linkous et al., 2019; Sun et al., 2025). Brain organoids composed of relatively pure cortical cholinergic neurons remain to be developed, though both transcription factor-based (this study) and small molecule-based (Muñoz et al., 2020) protocols for 2D cholinergic neurons are available. Limitations of the transcription factor-based approaches include a lack of natural stepwise differentiation processes and brain region specificity, but they are generally recognized to have increased purity and speed of maturation. Our study thus provides proof of principle for the potential of employing diverse human stem cell-derived systems for studying interactions between cancer and the nervous system.

METHODS

Human GBO culture

All experiments involving human patient-derived tissues were approved by the Institutional Review Board at the University of Pennsylvania. Patient-derived GBOs were generated and cultured as described previously (Jacob et al., 2020). A list of GBOs and hiPSC lines and their associated experiments are listed in Table S1.

hiPSC-derived cholinergic neuron culture and neuron-GBM co-culture

Human iPSC-derived cholinergic neurons were obtained from Elixirgen (CH-SeV-CW50065 and CH-SeV-CW50023). These iPSCs were derived from a healthy, 74-year-old Caucasian female donor (CW50065) or a healthy, 69-year-old Caucasian male donor (CW50023). Human iPSCs were differentiated into a cholinergic lineage via a transcription factor-based Sendai virus delivery approach (Elixirgen). Sterility (by direct immersion in liquid culture) and mycoplasma testing (by PCR) were performed for each batch (Elixirgen). Cells were plated at either 25,000 or 50,000 cells per well in a 24-well plate and maintained for the first 7 days according to manufacturer's protocol (Elixirgen). Beyond 7 days, cholinergic neurons were cultured in maintenance medium (Elixirgen, CH-MM) with 2X the recommended concentration of component P. For all neuron-GBM co-culture experiments, GBOs were dissociated into single cells as previously described (Sun et al., 2025) and seeded onto cholinergic neuron-containing coverslips.

Monosynaptic tracing

For monosynaptic tracing with cholinergic neurons, GBOs pre-labeled with EnvA G-deleted EGFP rabies virus as described previously (Sun et al., 2025) were dissociated and seeded onto coverslips at either a 1:50 or 1:100 tumor cell to neuron ratio. Coverslips were fixed for immunohistochemistry 3 days after seeding of GBM cells.

EM

For EM, cholinergic neurons (CW50065) were cultured until 8 days *in vitro*, upon which UP-10072 GBM cells were seeded for an additional 6 days. Subsequently, coverslips were briefly washed in 0.1 M phosphate buffer (PB) and fixed by immersion in 0.1 M PB with 4% PFA and 2% glutaraldehyde. They were then kept in this fixative at 4°C in contact lens holders until processing for EM on a 120 kV Talos transmission electron microscope.

scRNA-seq

For scRNA-seq experiments, at a cholinergic neuron (CW50065) age of 3 weeks *in vitro*, UP-10072 GBM cells were seeded into plates at a 1:20 tumor cell to neuron ratio for 6 days prior to dissociation for sequencing. We employed four separate conditions: (1) GBM-neuron co-culture, (2) GBM with neuronal conditioned media, (3) GBM cells alone, and (4) neurons alone. For the conditioned media

condition, media collected from cholinergic neuron cultures from the previous week was used to culture tumor cells alone for the period of 6 days. For all conditions, cells were dissociated via a previously published protocol (https://doi.org/10.17504/protocols.io.bh32j8qe) from $n = 4$ distinct coverslips prior to deep sequencing as previously described (Sun et al., 2025). Data processing and analysis procedures are described in the supplemental methods.

Cell proliferation and motility analyses

GBO cells were seeded into plates at a 1:10 tumor-to-neuron ratio at a cholinergic neuron age of 4 weeks *in vitro*. After 4 days, UP-10072-RTG co-cultures and controls were taken for live imaging on a confocal microscope (Zeiss LSM 710) for 16 h. Displacement (in μm/h from the original cell location) was obtained by the "Manual Tracking" plugin in Fiji. The same coverslips were fixed for immunohistochemistry for proliferation analyses. For proliferation analyses with inhibitors, simvastatin (MedChemExpress, HY-17502) or SH-42 (MedChemExpress, HY-143228) were added to co-cultures during the 4-day period to a final concentration of either 5 μM or 10 μM, respectively, prior to fixation for immunocytochemistry.

Experimental design and statistical analyses

Statistical analyses were conducted in R (v.4.3.1), with details on tests, sample sizes, and p values provided in the figure legends. Data in bar plots are presented as mean ± SEM, and, for boxplots, the center line represents the median, box edges show the 25th and 75th percentiles, and whiskers extend to maximum and minimum values. Statistical significance was defined as $p < 0.05$, with significance levels indicated as follows: ns for $p \geq 0.05$, $*p < 0.05$, $**p < 0.01$, and $***p < 0.001$. Data management principles (FAIR and CARE) were followed.

RESOURCE AVAILABILITY

Lead contact

Requests for further information and reagents may be directed to and will be fulfilled by lead contact Hongjun Song (shongjun@pennmedicine.upenn.edu).

Materials availability

GBOs generated in this study have been deposited within the University of Pennsylvania Brain Tumor Bank.

Data and code availability

Data reported in this paper will be shared by the lead contact upon request. This paper does not report original code. Any additional information required to reanalyze the data reported in this paper is available from the lead contact upon request. scRNA-seq data are deposited at NCBI Gene Expression Omnibus under accession number GSE294747.

ACKNOWLEDGMENTS

We thank the patients and their families for the generous donations of tissue specimens, A. Angelucci and G. Alepa for laboratory support; and A. Morschauser and the Penn Cell Sorting Laboratory for help with single-cell sorting. This work was supported by the National Institutes of Health (R35NS116843 to H.S., R35NS137480 to G.-l.M., and F31NS137664 to Y.S.), the Dean's Innovation Fund and the Institute for Regenerative Medicine at University of Pennsylvania (to H.S.), Dr. Miriam and Sheldon G. Adelson Medical Research Foundation (to G.-l.M.), and the Swiss National Science Foundation (to N.T.).

AUTHOR CONTRIBUTIONS

Y.S. led the study and performed most of the analyses. Y.S., X.W., and Z.Z. performed neuronal and GBO culture. N.T. contributed to electron microscopy experiments. K.H.P., Y.W., Y.F., D.Y.Z., F.Z., and K.M.C. contributed to additional analyses. E.L.-L.P., M.P. N., Z.A.B., and D.M.O. contributed to patient tissue collection. Y.S., X.W., Z.Z., G.-l.M., and H.S. conceived the study, designed experiments, and wrote the manuscript with input from all authors.

DECLARATION OF INTERESTS

G.-l.M. is on the advisory board of Stem Cell Reports.

SUPPLEMENTAL INFORMATION

Supplemental information can be found online at https://doi.org/10.1016/j.stemcr.2025.102534.

Received: February 10, 2025
Revised: May 17, 2025
Accepted: May 20, 2025
Published: June 19, 2025

REFERENCES

Clayton, B.L.L., Barbar, L., Sapar, M., Kalpana, K., Rao, C., Migliori, B., Rusielewicz, T., NYSCF Global Stem Cell Array® Team, Paull, D., Brenner, K., et al. (2024). Patient iPSC models reveal glia-intrinsic phenotypes in multiple sclerosis. Cell Stem Cell *31*, 1701–1713.e8.

Fuentes-Fayos, A.C., G-García, M.E., Pérez-Gómez, J.M., Montero-Hidalgo, A.J., Martín-Colom, J., Doval-Rosa, C., Blanco-Acevedo, C., Torres, E., Toledano-Delgado, Á., Sánchez-Sánchez, R., et al. (2023). Metformin and simvastatin exert additive antitumour effects in glioblastoma via senescence-state: clinical and translational evidence. EBioMedicine *90*, 104484.

Hsieh, A.L., Ganesh, S., Kula, T., Irshad, M., Ferenczi, E.A., Wang, W., Chen, Y.C., Hu, S.H., Li, Z., Joshi, S., et al. (2024). Widespread Neuroanatomical Integration and Distinct Electrophysiological Properties of Glioma-Innervating Neurons. Proc. Natl. Acad. Sci. USA *121*, e2417420121.

Jacob, F., Salinas, R.D., Zhang, D.Y., Nguyen, P.T.T., Schnoll, J.G., Wong, S.Z.H., Thokala, R., Sheikh, S., Saxena, D., Prokop, S., et al. (2020). A Patient-Derived Glioblastoma Organoid Model and Biobank Recapitulates Inter- and Intra-tumoral Heterogeneity. Cell *180*, 188–204.e22.

Krieger, T.G., Tirier, S.M., Park, J., Jechow, K., Eisemann, T., Peterziel, H., Angel, P., Eils, R., and Conrad, C. (2020). Modeling glioblastoma invasion using human brain organoids and single-cell transcriptomics. Neuro Oncol. *22*, 1138–1149.

Linkous, A., Balamatsias, D., Snuderl, M., Edwards, L., Miyaguchi, K., Milner, T., Reich, B., Cohen-Gould, L., Storaska, A., Nakayama, Y., et al. (2019). Modeling Patient-Derived Glioblastoma with Cerebral Organoids. Cell Rep. *26*, 3203–3211.e5.

Monje, M., Borniger, J.C., D'Silva, N.J., Deneen, B., Dirks, P.B., Fattahi, F., Frenette, P.S., Garzia, L., Gutmann, D.H., Hanahan, D., et al. (2020). Roadmap for the Emerging Field of Cancer Neuroscience. Cell *181*, 219–222.

Muñoz, S.S., Engel, M., Balez, R., Do-Ha, D., Cabral-da-Silva, M.C., Hernández, D., Berg, T., Fifita, J.A., Grima, N., Yang, S., et al. (2020). A Simple Differentiation Protocol for Generation of Induced Pluripotent Stem Cell-Derived Basal Forebrain-Like Cholinergic Neurons for Alzheimer's Disease and Frontotemporal Dementia Disease Modeling. Cells *9*, 2018.

Neftel, C., Laffy, J., Filbin, M.G., Hara, T., Shore, M.E., Rahme, G.J., Richman, A.R., Silverbush, D., Shaw, M.L., Hebert, C.M., et al. (2019). An Integrative Model of Cellular States, Plasticity, and Genetics for Glioblastoma. Cell *178*, 835–849.e21.

Nguyen, T.P., Wang, W., Sternisha, A.C., Corley, C.D., Wang, H.Y. L., Wang, X., Ortiz, F., Lim, S.K., Abdullah, K.G., Parada, L.F., et al. (2023). Selective and brain-penetrant lanosterol synthase inhibitors target glioma stem-like cells by inducing 24(S),25-epoxycholesterol production. Cell Chem. Biol. *30*, 214–229.e18.

Sun, Y., Wang, X., Zhang, D.Y., Zhang, Z., Bhattarai, J.P., Wang, Y., Park, K.H., Dong, W., Hung, Y.F., Yang, Q., et al. (2025). Brain-wide neuronal circuit connectome of human glioblastoma. Nature *641*, 222–231.

Tetzlaff, S.K., Reyhan, E., Layer, N., Bengtson, C.P., Heuer, A., Schroers, J., Faymonville, A.J., Langeroudi, A.P., Drewa, N., Keifert, E., et al. (2024). Characterizing and targeting glioblastoma neuron-tumor networks with retrograde tracing. Cell *188*, 390–411.

Thomas, E.A., Hsu, H.H., Griffin, M.T., Hunter, A.L., Luong, T., and Ehlert, F.J. (1992). Conversion of N-(2-chloroethyl)-4-piperidinyl diphenylacetate (4-DAMP mustard) to an aziridinium ion and its interaction with muscarinic receptors in various tissues. Mol. Pharmacol. *41*, 718–726.

Venkataramani, V., Tanev, D.I., Strahle, C., Studier-Fischer, A., Fankhauser, L., Kessler, T., Körber, C., Kardorff, M., Ratliff, M., Xie, R., et al. (2019). Glutamatergic synaptic input to glioma cells drives brain tumour progression. Nature *573*, 532–538.

Venkatesh, H.S., Morishita, W., Geraghty, A.C., Silverbush, D., Gillespie, S.M., Arzt, M., Tam, L.T., Espenel, C., Ponnuswami, A., Ni, L., et al. (2019). Electrical and synaptic integration of glioma into neural circuits. Nature *573*, 539–545.

Wang, X., Sun, Y., Zhang, D.Y., Ming, G., and Song, H. (2023). Glioblastoma modeling with 3D organoids: progress and challenges. Oxford Open Neuroscience *2*, kvad008.

Zhao, L., Qiu, Z., Yang, Z., Xu, L., Pearce, T.M., Wu, Q., Yang, K., Li, F., Saulnier, O., Fei, F., et al. (2024). Lymphatic endothelial-like cells promote glioblastoma stem cell growth through cytokine-driven cholesterol metabolism. Nat. Cancer *5*, 147–166.

Zhou, Y., Song, H., and Ming, G.L. (2024). Genetics of human brain development. Nat. Rev. Genet. *25*, 26–45. https://doi.org/10.1038/s41576-023-00626-5.

Stem Cell Reports, Volume *20*

Supplemental Information

Cholinergic neuron-to-glioblastoma synapses in a human iPSC-derived co-culture model

Yusha Sun, Xin Wang, Zhijian Zhang, Kristen H. Park, Yicheng Wu, Weifan Dong, Daniel Y. Zhang, Yao Fu, Feng Zhang, Zev A. Binder, Emily Ling-Lin Pai, MacLean P. Nasrallah, Kimberly M. Christian, Donald M. O'Rourke, Nicolas Toni, Guo-li Ming, and Hongjun Song

Cholinergic neuron-to-glioblastoma synapses in a human iPSC-derived co-culture model

Yusha Sun, Xin Wang, Zhijian Zhang, Kristen H. Park, Yicheng Wu, Weifan Dong, Daniel Y. Zhang, Yao Fu, Feng Zhang, Zev A. Binder, Emily Ling-Lin Pai, MacLean P. Nasrallah, Kimberly M. Christian, Donald M. O'Rourke, Nicolas Toni, Guo-li Ming, and Hongjun Song

Supplementary Information Inventory

Figures S1-4

Tables S1-3

Supplementary Methods

SUPPLEMENTARY FIGURES

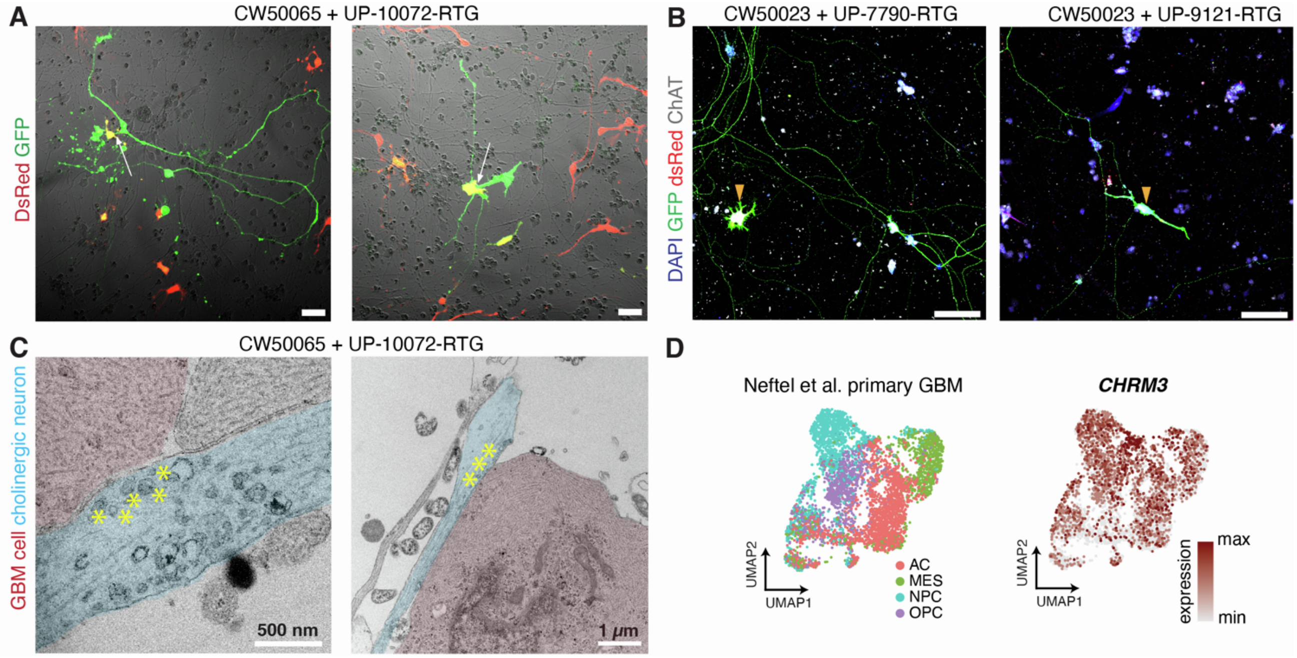

Figure S1. Extended characterization of human cholinergic neuron-to-glioblastoma synapses, related to Figures 1 and 2.
(A) Sample brightfield microscopy images overlaid with fluorescent channels at 2 days post co-culture for monosynaptic tracing experiments. Putative DsRed$^+$GFP$^+$ starter GBM cells are indicated with an arrow. The names of the hiPSC line and patient GBOs are listed. Scale bars, 50 μm.
(B) Sample confocal images of monosynaptic rabies spread from GFP$^+$DsRed$^+$ GBM cells to adjacent cholinergic neurons. Scale bar, 50 μm. Putative DsRed$^+$GFP$^+$ starter GBM cells are indicated with an arrow.
(C) Additional sample electron micrographs of morphological contacts between presynaptic cholinergic neurons (pseudo-colored blue) and postsynaptic GBM cells (pseudo-colored red) in co-culture. Yellow stars indicate synaptic vesicles. Scale bars as indicated, either 1 μm or 500 nm.
(D) Left, uniform manifold approximation and projection (UMAP) of Neftel et al. (Neftel et al., 2019) primary GBM cells, with assignment of transcriptional cellular states (see Methods). Right, expression feature plots for the CHRM3 receptor, indicating broad expression across cellular states in primary GBM.

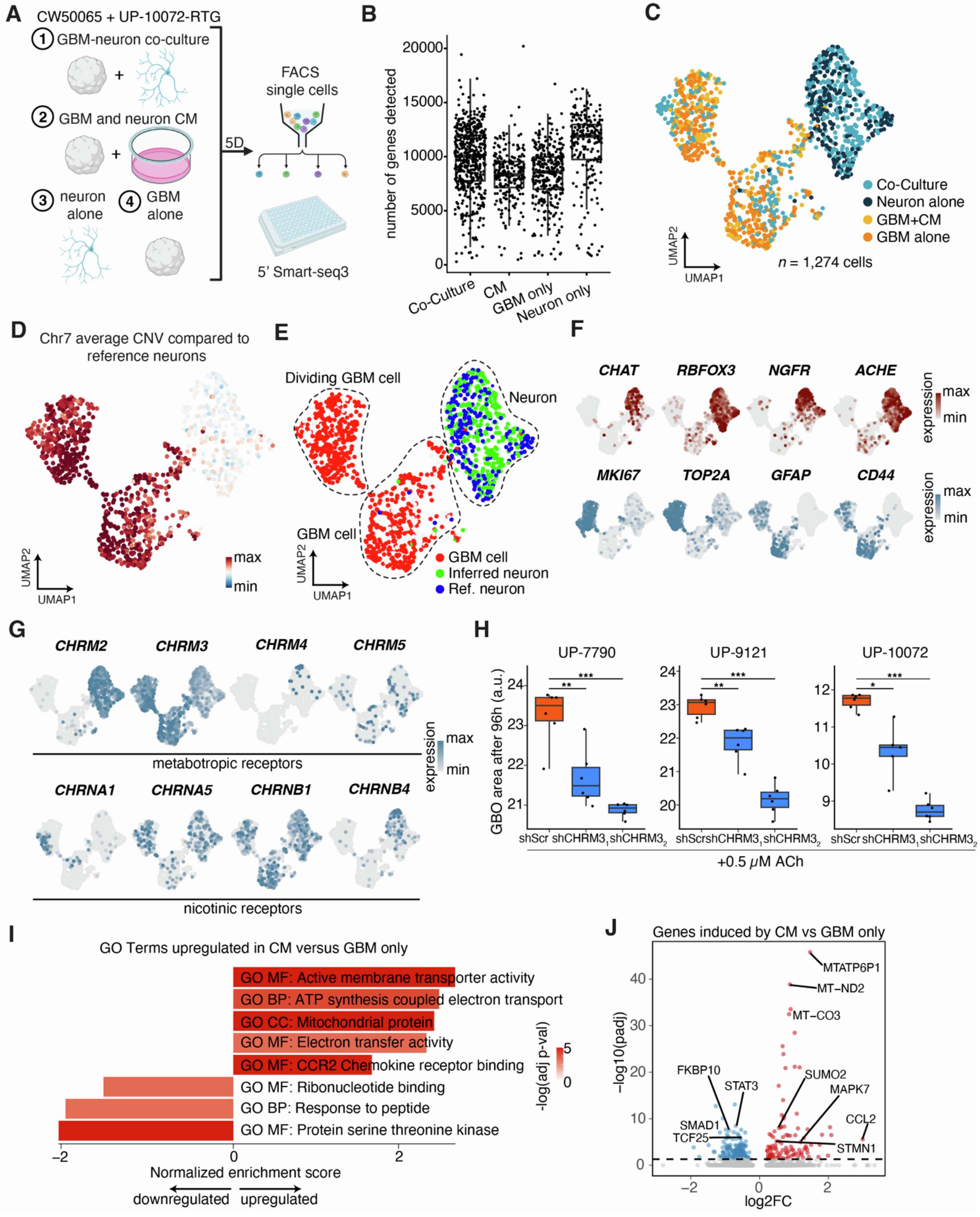

Figure S2. Single-cell transcriptional profiles of GBM cells and cholinergic neurons under different conditions, related to Figures 3 and 4.

(A) Schematic illustration of the scRNAseq experimental paradigm, consisting of either GBM-neuron co-culture, GBM cells with neuronal conditioned media (CM), neurons alone, or GBM alone cultures.
(B) Box plot of the number of genes detected from each culture condition, with each dot representing one cell.
(C-E) UMAP plots of n = 1,274 single cells colored by culture identity (C), average chromosome 7 copy number variation (CNV) (D), or by final assigned identity (E). The neuron-only culture condition (e.g., 'ref. neuron') was used as a reference to assign CNVs for the rest of the cells.
(F) Gene expression feature plots of representative cholinergic neuron genes (top) and genes expressed in malignant cells (bottom).
(G) Expression feature plots of various metabotropic and nicotinic acetylcholine receptors showing increased expression of CHRM3 across malignant cells.
(H) Boxplots of 2D measurement of areas of GBOs transduced with either a scrambled shRNA or two distinct shRNAs targeting CHRM3 cultured for 96 hours, showing decreased tumor cell viability and proliferation in the presence of 0.5 μM ACh (n = 5-6 organoids per condition; UP-7790: **p=0.0068 (shScr vs. $shCHRM3_1$), ***p=0.0006 (shScr vs. $shCHRM3_2$); UP-9121: **p=0.0046 (shScr vs. $shCHRM3_1$), ***$p=1.2\times10^{-6}$ (shScr vs. $shCHRM3_2$); UP-10072: *p=0.024 (shScr vs. $shCHRM3_1$), ***$p=8.2\times10^{-9}$ (shScr vs. $shCHRM3_2$); Welch's t-tests with Bonferroni's correction for multiple comparisons).
(I-J) Representative GO terms (I) and volcano plots (J) of genes induced by CM by comparing tumor cells in the CM condition versus GBM alone, similar to Figures 4E-4F.

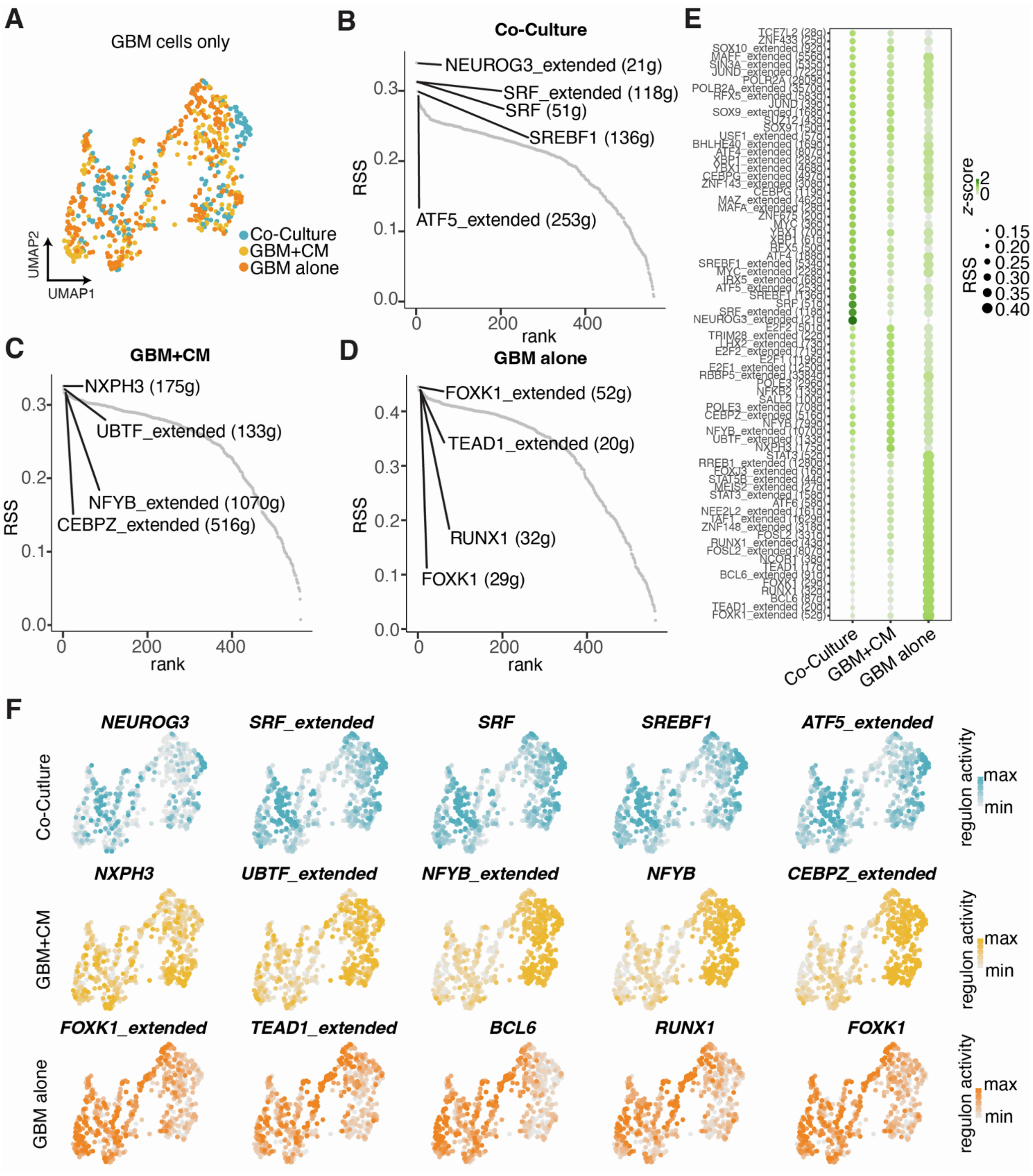

Figure S3. Transcriptional regulators of GBM cells in the presence of cholinergic neurons identified by gene regulatory network (GRN) analyses, related to Figures 3 and 4.
(A) UMAP plots of *n* = 672 single cells confidently assigned to malignant identity, colored by condition. CM, conditioned media.
(B-D) Regulon specificity scores in co-culture (B), CM (C), or GBM alone (D) conditions. Top motifs are indicated for each condition.
(E) Bubble plot of top regulons in each condition.
(F) Feature plots of the activity of top regulons for each condition as implemented in the SCENIC package (Aibar et al., 2017).

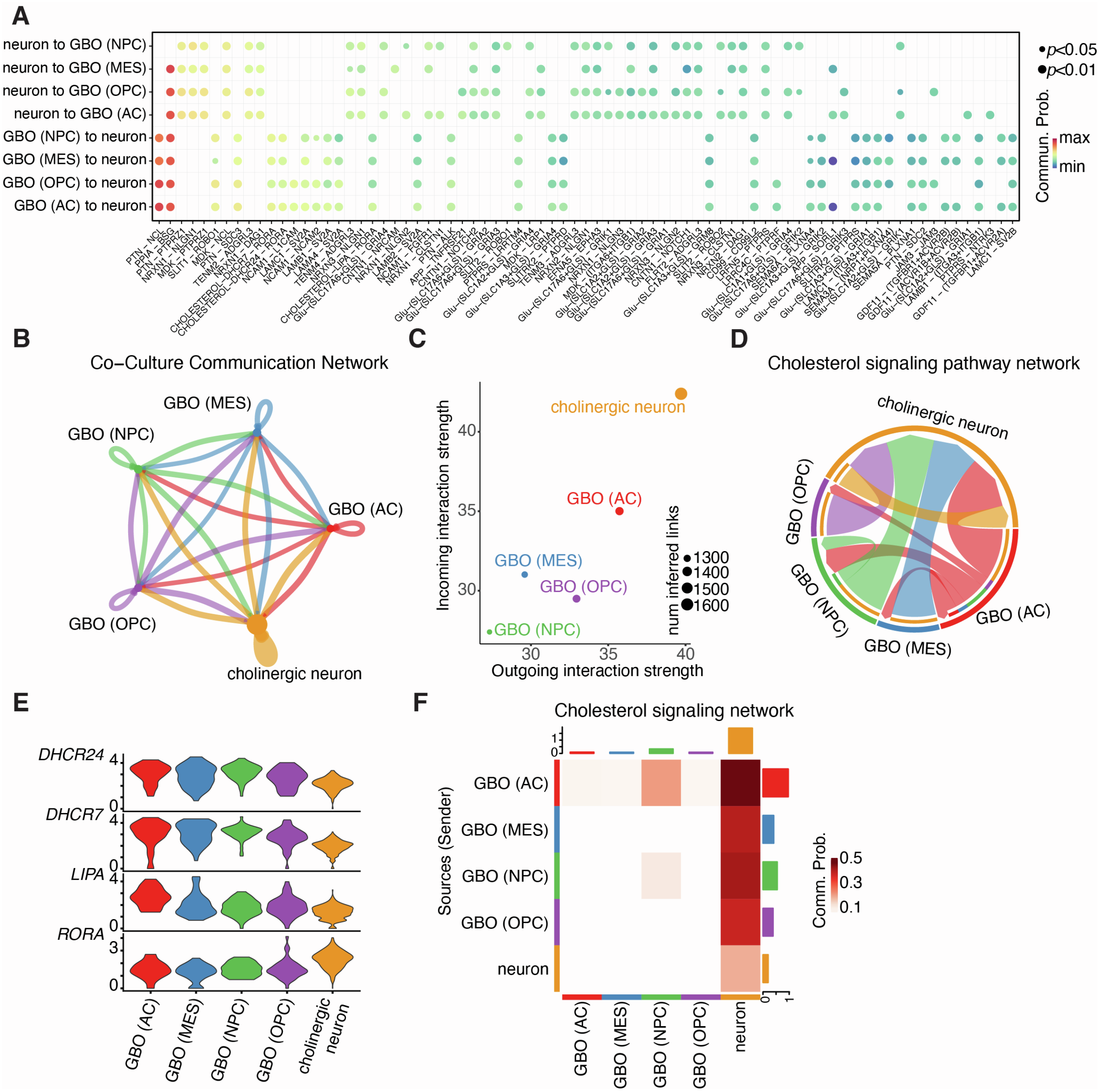

Figure S4. Major signaling pathways between cholinergic neurons and GBM cells of different states in co-culture identified by cell-cell interaction analyses, related to Figures 3 and 4.
(A) Bubble plot of significant inferred cell-cell interactions between cholinergic neurons and AC-like, OPC-like, NPC-like, or MES-like GBM cells.
(B) Circle diagram of aggregated cell-cell communication network between GBM cells of different states and neurons.
(C) Visualization of dominant senders and receivers in co-culture based on number of inferred links.
(D-F) Analyses highlighting the importance of the cholesterol metabolism pathway in GBM-cholinergic neuron interactions via a chord diagram (D), violin plots of relevant pathway genes (E), and heatmap of the cholesterol signaling network (F).

SUPPLEMENTARY TABLES (in Excel)

Table S1. GBO and hiPSC line additional information, related to Figures 1-4.
Flow-chart of experiments performed in the study and the associated GBO line(s) and/or hiPSC line(s) used to derive cholinergic neurons as applicable.

Table S2. Differentially expressed genes in GBM cells under different conditions, related to Figures 3-4.
Lists of differentially expressed genes between GBM cells in co-culture versus conditioned media, co-culture versus tumor alone, or conditioned media versus tumor alone.

Table S3. Primer sequences for shRNA targeting, related to Figure S2.
Oligonucleotide sequences for plasmid construction for knockdown of CHRM3. Sequences for CHRM3 KD 2 and Scramble were previously reported in (Sun et al., 2025).

SUPPLEMENTARY METHODS

Immunohistochemistry

For immunohistochemistry, coverslips were washed 2X with ice-cold DPBS, gently fixed for 20 minutes using ice-cold 4% paraformaldehyde (PFA), and washed 2X again with DPBS, prior to incubation with blocking buffer containing (TBS with 0.1% Tween-20 (Sigma-Aldrich, T8787-50ML), 0.5% Triton X-100, 10% donkey serum (Millipore, S30), 1% BSA (Sigma-Aldrich, B6917), and 22.52 mg/mL glycine (Sigma-Aldrich, 50046-50G) for 1 hour at room temperature (RT). Coverslips were then incubated in diluted primary antibodies in antibody buffer (TBS with 0.1% Tween-20, 0.5% Triton X-100, and 5% donkey serum) overnight at 4°C. The next day, coverslips were washed 3X in TBST (TBS with 0.1% Tween-20) for 5 minutes each and incubated with secondary antibodies diluted in antibody buffer as described above for 1-2 hours at RT. Where applicable, DAPI (Thermo Fisher Scientific, D1306, 1:500) was added during the secondary antibody incubation. Coverslips were washed 3X in TBST for 5 minutes each and then mounted on a glass slide (Thermo Fisher Scientific, 1518848) in mounting medium (Vector Laboratories, H-1000-10) and sealed with nail polish prior to confocal imaging (Zeiss LSM 810 or Zeiss LSM 710) as *z*-stacks with either 10X, 20X, or 40X objectives.

The following primary antibodies were used: Goat anti-RFP (Biorbyt, orb11618, 1:500), rabbit anti-RFP (Rockland, 600-401-379,1:500), chicken anti-GFP (Abcam, ab13970, 1:2000), goat anti-GFP (Rockland, 600-101-215, 1:500), goat anti-ChAT (Sigma-Aldrich, AB144P-200UL, 1:200), rabbit anti-VAChT (Synaptic Systems, 139103, 1:500), mouse anti-KI67 (BD Biosciences, 550609, 1:500), rabbit anti-KI67 (Abcam, ab16667, 1:500), mouse anti-NeuN (Thermo Fisher Scientific, MA5-33103, 1:500), and chicken anti-beta-microtubules 3 (Aves, TUJ-0020, 1:1000). The following secondary antibodies were used: donkey anti-chicken Alexa Fluor 488 (Thermo Fisher Scientific, A-78948, 1:500), donkey anti-mouse Alexa Fluor 555 (Thermo Fisher Scientific, A-31570, 1:500), donkey anti-goat Alexa Fluor 647 (Thermo Fisher Scientific, A-21447, 1:500), donkey anti-rabbit Alexa Fluor 555 (Thermo Fisher Scientific, A-31572, 1:500), donkey anti-mouse Alexa Fluor 647 (Thermo Fisher Scientific, A-31571, 1:500), and donkey anti-rabbit Alexa Fluor 405 (Thermo Fisher Scientific, A-48258, 1:500).

Viral vectors and plasmids

EnvA-pseudotyped G-deleted EGFP rabies virus was purchased from the Salk viral core (32635). The GBOs expressing the retroviral RTG helper plasmid (Addgene #235698) were generated as described previously (Sun et al., 2025). The UP-10072 GBOs expressing the red-shifted calcium indicator jRGECO1α were generated as described previously (Dana et al., 2016; Sun et al., 2025). The lentiviral construct for optogenetic depolarization of cholinergic neurons was generated using

the pLenti-EF1a-hChR2(H134R)-EYFP-WPRE plasmid (Addgene, 20942). shRNAs were generated as described previously (Sun et al., 2025), with targeting sequences listed in Table S3.

Calcium imaging and analyses

For Ca^{2+} imaging of co-cultures, pure cholinergic neurons (CW50065) were infected with lenti-ChR2 at 3 days *in vitro* (DIV). At 4 weeks *in vitro*, UP-10072 GBO cells expressing jRGECO1α were seeded into plates at a 1:20 tumor cell to neuron ratio for Ca^{2+} imaging. Three days after seeding, live Ca^{2+} imaging was performed using a confocal microscope (Zeiss LSM 710) with a 10X objective by acquiring images at 2 Hz in the 555 nm wavelength channel. Light pulses for optogenetic stimulation of cholinergic neurons were delivered by a laser (LRD-0470-PFFD-00100-05) with 470 nm wavelength (~0.32 mW/mm^2 power) connected to a power supply (PSU-H-LED), with pulse length and frequency set by a programmable pulse generator (Master 8). Simultaneous Ca^{2+} imaging and optogenetic stimulation (10 ms pulses at 20 Hz for 10 seconds) were performed with or without the presence of 100 μM 4-DAMP.

In brief, recordings were exported as .CZI files and imported to ImageJ/FIJI for quantification. Cells that exhibited Ca^{2+} transients in response to optogenetic stimulation were analyzed. To generate the dF/F traces, we first obtained the baseline intensity trace by computing the tenth percentile of a moving 50-frame window of each raw trace using the rollapply function in R. The dF/F trace was then defined as the $\frac{\text{raw intensity} - \text{baseline intensity}}{\text{baseline intensity}}$ at each timepoint. The trace was then smoothed twice (triangular moving average) via a 7-frame window. We defined $\Delta dF/F_{max}$ as the maximum change in dF/F between the mean dF/F in a 10-second window prior to light stimulation compared to maximal dF/F.

Post-fixation processing for electron microscopy

Coverslips then washed 3X for 5 minutes each in 0.1 M PB and postfixed 1h in 1% osmium tetroxide in 0.1 M PB. They were then washed 3X for 5 minutes each in 0.1M PB, then in double distilled water for 1 minute and dehydrated in an ascending series of ethanol (50%, 70%, 90%, 100%), 7 minutes each, followed by acetone 100% 3X for 7 minutes each. Cells were then cured in epoxy resin:acetone mix (1:1; 2:1) for 30 minutes each, and epoxy 100% for 10 minutes. Coverslips were removed from the holders and placed face up on a glass slide and cured for 1h at 50°C. After this, a plastic capsule filled with resin was placed on top of each coverslip and cured for 48h at 60°C. The coverslips were removed from the blocks by plunging into liquid nitrogen. Blocks were then trimmed, and serial sections (50nm thickness) were collected on a single-slot formvar-coated copper grid. Sections were contrasted with uranyl acetate and lead citrate and observed at the

electron microscopy facility of the University of Lausanne, on a 120KV Talos transmission electron microscope at a magnification of 8500x.

scRNAseq data processing and analysis

Single cell RNA sequencing data were processed as previously described (Sun et al., 2025) prior to analysis. Count matrices generated using the “GeneFull” option in STARsolo, which includes intronic counts, were imported into R (v4.3.1) using the Seurat package (v4.3.0.1). Cells containing fewer than 1000 UMIs or more than 20% mitochondrial UMIs were excluded. Data normalization was performed using SCTransform with vst.flavor = "v2", variable.features.n = 15000, and regression on mitochondrial UMIs percentage and total UMI counts. Copy number aberration (CNA) analysis was conducted using the HoneyBADGER R package (Fan et al., 2018). GBM cellular states were assigned as previously described (Neftel et al., 2019), utilizing the get.sig.scores function (LeBlanc et al., 2022). Primary patient GBM transcriptional data (Neftel et al., 2019) were integrated with harmony (Korsunsky et al., 2019). Differentially expressed genes (DEGs) were identified using Seurat’s FindAllMarkers function, applying a log fold-change threshold of 0.1 and an adjusted *p*-value < 0.05 (Wilcoxon rank-sum test with Bonferroni correction). Gene Ontology (GO) analyses were performed using fgsea package in R (Korotkevich et al., 2021). Gene regulatory network analyses were conducted with SCENIC in R (Aibar et al., 2017) and cell-cell interaction analyses were performed with CellChat v2 (Jin et al., 2025) with default parameters.

Additional references

Aibar, S., González-Blas, C.B., Moerman, T., Huynh-Thu, V.A., Imrichova, H., Hulselmans, G., Rambow, F., Marine, J.-C., Geurts, P., Aerts, J., et al. (2017). SCENIC: single-cell regulatory network inference and clustering. Nat Methods *14*, 1083–1086. https://doi.org/10.1038/nmeth.4463.

Dana, H., Mohar, B., Sun, Y., Narayan, S., Gordus, A., Hasseman, J.P., Tsegaye, G., Holt, G.T., Hu, A., Walpita, D., et al. (2016). Sensitive red protein calcium indicators for imaging neural activity. eLife *5*, e12727. https://doi.org/10.7554/eLife.12727.

Fan, J., Lee, H.-O., Lee, S., Ryu, D., Lee, S., Xue, C., Kim, S.J., Kim, K., Barkas, N., Park, P.J., et al. (2018). Linking transcriptional and genetic tumor heterogeneity through allele analysis of single-cell RNA-seq data. Genome Res. gr.228080.117. https://doi.org/10.1101/gr.228080.117.

Jin, S., Plikus, M.V., and Nie, Q. (2025). CellChat for systematic analysis of cell–cell communication from single-cell transcriptomics. Nat Protoc *20*, 180–219. https://doi.org/10.1038/s41596-024-01045-4.

Korotkevich, G., Sukhov, V., Budin, N., Shpak, B., Artyomov, M.N., and Sergushichev, A. (2021). Fast gene set enrichment analysis. 060012. https://doi.org/10.1101/060012.

Korsunsky, I., Millard, N., Fan, J., Slowikowski, K., Zhang, F., Wei, K., Baglaenko, Y., Brenner, M., Loh, P., and Raychaudhuri, S. (2019). Fast, sensitive and accurate integration of single-cell data with Harmony. Nat Methods *16*, 1289–1296. https://doi.org/10.1038/s41592-019-0619-0.

LeBlanc, V.G., Trinh, D.L., Aslanpour, S., Hughes, M., Livingstone, D., Jin, D., Ahn, B.Y., Blough, M.D., Cairncross, J.G., Chan, J.A., et al. (2022). Single-cell landscapes of primary glioblastomas and matched explants and cell lines show variable retention of inter- and intratumor heterogeneity. Cancer Cell *40*, 379-392.e9. https://doi.org/10.1016/j.ccell.2022.02.016.

Neftel, C., Laffy, J., Filbin, M.G., Hara, T., Shore, M.E., Rahme, G.J., Richman, A.R., Silverbush, D., Shaw, M.L., Hebert, C.M., et al. (2019). An Integrative Model of Cellular States, Plasticity, and Genetics for Glioblastoma. Cell *178*, 835-849.e21. https://doi.org/10.1016/j.cell.2019.06.024.

Sun, Y., Wang, X., Zhang, D.Y., Zhang, Z., Bhattarai, J.P., Wang, Y., Park, K.H., Dong, W., Hung, Y.-F., Yang, Q., et al. (2025). Brain-wide neuronal circuit connectome of human glioblastoma. Nature 1–3. https://doi.org/10.1038/s41586-025-08634-7.
